# Molecular Analysis of the E2F/DP Gene Family of *Daucus carota* and Involvement of the DcE2F1 Factor in Cell Proliferation

**DOI:** 10.3389/fpls.2021.652570

**Published:** 2021-03-12

**Authors:** Lara Perrotta, Roberta Giordo, Dennis Francis, Hilary J. Rogers, Diego Albani

**Affiliations:** ^1^Department of Agricultural Sciences, University of Sassari, Sassari, Italy; ^2^School of Biosciences, Cardiff University, Cardiff, United Kingdom

**Keywords:** cell proliferation, *Daucus carota*, E2F/DP genes, ectopic expression, gene expression, polycotyly

## Abstract

E2F transcription factors are key components of the RB/E2F pathway that, through the action of cyclin-dependent kinases, regulates cell cycle progression in both plants and animals. Moreover, plant and animal E2Fs have also been shown to regulate other cellular functions in addition to cell proliferation. Based on structural and functional features, they can be divided into different classes that have been shown to act as activators or repressors of E2F-dependent genes. Among the first plant E2F factors to be reported, we previously described DcE2F1, an activating E2F which is expressed in cycling carrot (*Daucus carota*) cells. In this study, we describe the identification of the additional members of the E2F/DP family of *D. carota*, which includes four typical E2Fs, three atypical E2F/DEL genes, and three related DP genes. Expression analyses of the carrot *E2F* and *DP* genes reveal distinctive patterns and suggest that the functions of some of them are not necessarily linked to cell proliferation. DcE2F1 was previously shown to transactivate an E2F-responsive promoter in transient assays but the functional role of this protein *in planta* was not defined. Sequence comparisons indicate that DcE2F1 could be an ortholog of the AtE2FA factor of *Arabidopsis thaliana*. Moreover, ectopic expression of the *DcE2F1* cDNA in transgenic Arabidopsis plants is able to upregulate *AtE2FB* and promotes cell proliferation, giving rise to polycotyly with low frequency, effects that are highly similar to those observed when over-expressing *AtE2FA*. These results indicate that DcE2F1 is involved in the control of cell proliferation and plays important roles in the regulation of embryo and plant development.

## Introduction

E2F transcription factors are key components of the RB/E2F pathway which is controlled by cyclin-dependent kinases and regulates cell cycle progression in both plants and animals. The primary role of the RB/E2F pathway in cell proliferation is confirmed by its disruption in most human cancers (Kent and Leone, 2019). Nevertheless, additional roles of the E2F factors have been emerging, and both plant and animal E2Fs regulate other cellular functions in addition to cell cycle progression (Dimova and Dyson, 2005; Fajas, 2013; Chandran et al., 2014; Wang et al., 2014; Taylor-Teeples et al., 2015; Horvath et al., 2017). From a structural and functional point of view, E2Fs can be divided into different classes and can act as activators or repressors of E2F-dependent genes. In both plant and animals, the E2F/DP family of transcription factors includes typical E2Fs and their related dimerizing partners, called DPs, as well as atypical E2Fs, named also DEL (DP-E2F-Like) in plants (Lammens et al., 2009). The typical E2Fs and the DP proteins possess a homologous DNA-binding domain and bind together to the DNA, forming heterodimers thanks to the presence of a leucine zipper in their dimerizing region. The atypical E2F/DEL factors, instead, lack dimerizing regions and do not interact with DPs, but feature a duplication of the homologous DNA-binding domain that allows them to bind DNA autonomously (Mariconti et al., 2002). Moreover, the typical E2Fs, but not the DPs or the atypical E2F/DEL proteins, possess a conserved C-terminal transactivating domain that allows the transcriptional activation of the E2F gene targets. The transactivating domain also includes a short region recognized by the RB tumor suppressor and by other “pocket proteins” whose binding abolishes transcriptional activation and confers instead strong repressive functions (Dimova and Dyson, 2005). Therefore, based on their interaction with pocket proteins, the typical E2Fs can act either as activators or as repressors of gene expression, whereas the atypical E2Fs, which lack transactivating domains, are not able to activate their target genes but are believed to downregulate E2F-dependent gene expression by competing for DNA binding with the typical E2Fs.

According to the canonical RB/E2F pathway, in quiescent cells, the typical E2Fs are complexed with hypophosphorylated pocket proteins and can repress genes involved in cell proliferation. Upon mitogenic induction of cell proliferation, the phosphorylation of the pocket proteins by cyclin-dependent kinases (CDKs) allows the release of free E2Fs that can activate the expression of S-phase genes and promote cell cycle progression (Rubin et al., 2020). Thus, the repressive or activating functions of the typical E2Fs allow a concerted regulation of the cell cycle genes and of other target genes, although the expression of these genes can be controlled also by the atypical E2Fs that act as competitive inhibitors of the activating E2Fs.

The E2F/DP family of *Arabidopsis thaliana*, the best characterized so far in plants, includes three typical E2Fs (AtE2FA, AtE2FB, and AtE2FC), three atypical E2Fs (AtE2FD/DEL2, AtE2FE/DEL1, and AtE2FF/DEL3), and two DPs (AtDPA and AtDPB; Mariconti et al., 2002). Functional studies have revealed specific roles and synergistic or antagonistic interplays of the Arabidopsis E2Fs. Moreover, except for *AtE2FA*, the promoters of all the other AtE2F genes contain putative E2F binding sites and cross-regulation of various E2Fs has been described as part of a complex regulatory network in both plant and animals (Vandepoele et al., 2005; Sozzani et al., 2006, 2010; Berckmans et al., 2011a; Kent and Leone, 2019).

Concerning the roles of the Arabidopsis E2Fs, overexpression of *AtE2FA* together with *AtDPA* promotes mitosis and endoreduplication, strongly affecting plant development (De Veylder et al., 2002). However, the increased endoreduplication appears to occur only in differentiated undividing cells, and additional studies have revealed that AtE2FA is important for the maintenance of the root meristems, where in complex with RBR can repress DNA endoreduplication and cell differentiation (Magyar et al., 2012). Moreover, in an interplay between the E2F factors, AtE2FA is able to promote the expression of *AtE2FB*, whose overexpression is able to induce cell division and reduce endoreduplication (Sozzani et al., 2006). In meristematic cells, AtE2FB is released from RBR by the activity of CDKs and is believed to activate cell cycle genes, including the mitotic *CDKB1;1* that promotes cell proliferation (Magyar et al., 2005). However, in differentiated cells, AtE2FB is complexed with RBR, which does not allow the activation of cell cycle genes. Compared to AtE2FA and AtE2FB, that can either activate or repress their target genes, AtE2FC is believed to act mainly as a repressor of E2F-regulated genes in association with AtRBR and its overexpression reduces cell division and leads to increased endoreduplication (del Pozo et al., 2006). With respect to the atypical E2Fs, *AtE2FD/DEL2* overexpression has been reported to promote cell proliferation by inhibiting the expression of E2F target genes that encode repressors of cell division (Sozzani et al., 2010). The AtE2FE/DEL1 factor, instead, is able to reduce endoreduplication by repressing the APC/C activator CCS52A2, which is needed for the degradation of mitotic cyclins (Vlieghe et al., 2005). In contrast, the AtE2FF/DEL3 factor has been proposed to downregulate cell wall biogenesis genes that allow cell elongation after cell cycle exit (Ramirez-Parra et al., 2004).

Among the first plant E2F factors to be reported, we previously described DcE2F1, a typical E2F in carrot (*Daucus carota*) cells (Albani et al., 2000). DcE2F1 was shown to transactivate a reporter gene in transient assays but its functional role *in planta* was not defined. In this study, we describe the remaining members of the E2F/DP family of *D. carota*, that includes four typical E2Fs, three atypical E2F/DEL, and three DPs, and report a functional analysis of *DcE2F1*. Sequence comparisons revealed that DcE2F1 is likely to be the ortholog of the AtE2FA factor of *A. thaliana*. Moreover, the phenotypes observed in the Arabidopsis transformants ectopically expressing the *DcE2F1* cDNA are highly similar to those observed overexpressing *AtE2FA* and are consistent with a promotion of cell proliferation in embryos and young seedlings.

## Materials and Methods

### Plant Material

Seedlings of *D. carota* cv. Berlicum 2 were obtained from seeds which were surface sterilized for 8–10 h in 2% v/v PPM® (Plant Preservative Mixture, Plant Cell Technology) supplemented with 50 mg/L magnesium salts (MgSO_4_) and then germinated in Magenta boxes containing MS salts (Duchefa Biochemie), supplemented with Sucrose (10 g/L), and Phyto agar (8 g/L; Duchefa Biochemie). The seedlings were grown aseptically in a growth cabinet at 22°C under long day conditions of 16 h of light and 8 h of dark. To obtain taproot samples, some of the seedlings were transferred to soil and grown for 2 months under the same conditions.

Wild type or transgenic *A. thaliana* ecotype Columbia seeds were surface sterilized as the carrot seeds. Following imbibition for 3 days in 0.1% agarose at 4°C in the dark, the seeds were germinated on Petri plates using the same media and growth conditions as the carrot seedlings. For the root growth assay, the seeds were germinated on the same media placing them near one side of squared 12 cm plates that were incubated in a vertical position in the growth cabinet.

### Identification of Carrot E2F and DP Genes

To identify the carrot E2F and DP genes, TBLASTN searches were performed against the carrot genome of the variety Kuroda (Xu et al., 2014), reported in the CarrotDB database,^1^ and against the v2.0 release of the carrot genome from the doubled-haploid Nantes-type carrot (DH1; Iorizzo et al., 2016), available at the Phytozome platform.^2^ The BLAST searches were conducted using the DcE2F1 and the Arabidopsis E2F and DP protein sequences as queries. Additional searches were performed with the consensus DNA-binding domain sequence, which is conserved among the E2F and DP proteins. The exon regions were further validated, performing BLASTN searches against RNA-Seq data available for various *D. carota* organs, which are reported in the NCBI database under the accession SRP062159.

To obtain the cDNAs of the carrot E2F and DP transcripts, reverse transcription PCR (RT-PCR) reactions were performed on mRNAs isolated from a carrot cell culture and from carrot leaves of the cv. Lunga di Amsterdam which were pooled and reverse transcribed using the SuperScript® Reverse Transcriptase (Invitrogen) and oligo dT primers as reported previously (Albani et al., 2000). The coding regions of the E2F and DP genes were amplified by PCR performed with high fidelity Pfu DNA polymerase (Thermo Fisher Scientific) using forward primers overlapping the putative ATG start codons and reverse primers extending over the termination codons, in combination with internal primers. The resulting PCR fragments were sequenced to verify the correspondence to the genomic sequences. All the genes were mapped to chromosomes based on the information available on the Phytozome platform and a chromosomal location image was made using the online tool MapGene2Chromosome V2.^3^

### Sequence Alignment and Phylogenetic Analyses

Multiple alignments of the E2F and DP proteins from carrot and Arabidopsis were performed online using MUSCLE^4^ (Madeira et al., 2019) and publication-quality outputs of the sequence alignments were generated with BOXSHADE 3.21.^5^ he phylogenetic analyses were performed on the Phylogeny.fr platform (Dereeper et al., 2008). The MSA alignments obtained with MUSCLE (v3.8.31) were curated to remove ambiguous regions using Gblocks (v0.91b) with default settings. The phylogenetic trees were constructed using the maximum-likelihood method implemented in the PhyML program (v3.1), applying the WAG substitution model with default settings. Bootstrapping (*n* = 100) was performed to assess the reliability of the branching patterns. Graphical representations of the phylogenetic trees were generated online using iTOL.^6^

To confirm and expand the phylogenetic analyses, the sequences of the annotated E2F and DP proteins of coriander (*Coriandrum sativus*) were fetched from the Coriander Genome Database^7^ (Song et al., 2020). The phylogenetic analysis with the inclusion of the coriander E2F and DP proteins was conducted on the Galaxy/Pasteur platform^8^ (Mareuil et al., 2017) using MUSCLE (v3.8.31) to produce the multiple sequence alignment. After curation of the MSA with NOISY (v1.5.12.1), the maximum-likelihood phylogenetic trees were built with the FASTTREE program (v2.1.9) using an LG substitution model and bootstrapping with 1,000 replicates for branch support. The tree image was generated online using iTOL.^9^

### Nucleic Acids Extraction and qRT-PCR Analyses

Genomic DNA from transformed Arabidopsis T1 plants was isolated using a modified CTAB protocol as described previously (Maniga et al., 2017). To identify single insertion lines, quantitative PCR (qPCR) with DcE2F1 primers was performed on a BioRad iCycler iQ ™ machine using the Qiagen QuantiTect SYBR® Green PCR Kit, normalizing against the amplification of a portion of the Arabidopsis AtE2FF gene.

Total RNA extractions from carrot organs and Arabidopsis plants were performed using the Qiagen RNeasy mini-kit. To avoid DNA contamination, the RNA samples were processed with the Qiagen RNase-free DNase set during the extraction. RNA concentration and quality were verified by spectrophotometry using A260/A280 ratio and by electrophoresis on denaturing formaldehyde gels.

For quantitative reverse transcription PCR (qRT-PCR) analyses, 1 μg of RNA was reverse transcribed using the SuperScript® III Reverse Transcriptase (Invitrogen) with a combination of hexamers and oligo dT primers. The qRT-PCR analyses were repeated three times using independent biological replicates. qPCR was performed on a BioRad iCycler iQ ™ machine using the Qiagen QuantiTect SYBR® Green PCR Kit. Three technical replicates were used to analyze each sample, following the manufacturer’s recommended amplification conditions. The amplification of carrot actin RNA or 18S ribosomal RNA, whose sequence is highly conserved in both carrot and Arabidopsis, was used as a reference for normalization and quantification was calculated following the 2^−*Δ*ΔCT^ method (Livak and Schmittgen, 2001). The PCR primers were designed using the Primer3 online software^10^ and all their sequences are listed in Supplementary Table S1.

### Analysis of RNASeq Data

To analyze the expression profiles of the carrot E2F and DP transcripts in different organs, RNA-Seq data from the BioProject accession PRJNA291977 were downloaded from NCBI. Read libraries were specific from leaves at three stages of growth (0.5–1 cm young sprout, 2–2.5 cm leaves, and 7–8 cm leaves), mature leaf petioles, fibrous roots, flower buds, unopen flowers, open flowers, germinating seeds, or callus tissue. The individual accession numbers are reported in Supplementary Table S2. The size of the RNA-seq libraries ranged from 4.24 to 8.90 Gbases. The analyses were conducted on the Galaxy platform^11^ and to evaluate the expression of the carrot E2F and DP genes, transcripts per million (TPM) values were calculated using Salmon (v0.11.2) with default parameters. Differential expression analyses comparing to the stage 1 leaf were performed with the DESeq2 package (v2.11.38), and fold change cutoff of 2 and adjusted *p* ≤ 0.05 were taken as statistically significant threshold. The log2-transformed fold change values were used to generate heatmaps by the heatmapper server^12^ (Babicki et al., 2016).

### *In silico* Analysis of the DcE2F and DcDP Promoters

For the analysis of the presence of putative regulatory sites in the DcE2F and DcDP promoters, the sequence of the 1,000 bp region upstream to the ATG codons of each gene was extracted from the genome sequence available at Phytozome and searches of regulatory elements reported in literature for animal or plant E2F promoters were performed. The relevant *cis*-acting elements included the consensus sites for E2F (NNTSSCGSN), LBD (NNGCGGCWN), CSP4 (KTTTTWTTN), and PIF4 (NCACRTGNN), which is bound also by Myc proteins, and the G2-like site (NRGAATMTN) which is recognized by Myb factors. The map showing the location of the selected *cis*-elements in the DcE2F and DcDP promoters was created using the drawing tool of the Regulatory Sequence Analysis Tools (RSAT) platform.^13^

### Generation and Phenotypic Characterization of Transgenic Arabidopsis Lines Ectopically Expressing DcE2F1 or AtE2FA

To generate vectors for the ectopic expression of *DcE2F1* or *AtE2FA*, the corresponding cDNA fragments were inserted in the polylinker of the pFF19 vector (Timmermans et al., 1990), downstream of the double CaMV 35S promoter and upstream of the 35S terminator sequence. The entire cassette was then excised as a *Hin*dIII-*Eco*RI fragment and cloned into the pCambia 1,304 binary vector, which also contains the GUS reporter gene under the control of the CaMV 35S promoter. The transgenic Arabidopsis lines were generated by the floral dip method (Clough and Bent, 1998) using the *Agrobacterium tumefaciens* GV3101/pMP90 strain. Transformed T1 and progeny plants were selected on MS plates containing Hygromycin (10 mg/L) and after 2 weeks, the resistant plants were transferred to recovery plates without the selection agent and grown for 1 more week in aseptic conditions before transferring them to soil. Plants were grown to maturity in growth cabinets set at long day conditions of 16 h of light (22 ± 3°C) and 8 h of dark (22 ± 3°C), with 70% relative humidity. Single insertion lines were identified by qPCR reactions with leaf DNA and were confirmed by segregation of hygromycin resistance in the T2 progeny.

Differential interference contrast microscopy was used to examine cotyledons and root meristems of plantlets that had been fixed with FAA (50% ethanol, 5% acetic acid, and 10% formaldehyde) for 3 h, de-pigmented in increasing ethanol concentrations and cleared in chloral hydrate (8 g chloral hydrate, 1 ml glycerol, and 2 ml H_2_O). The analyses were performed on a Zeiss Aziophot microscope equipped with an Infinity1 camera (Lumenera) and the resulting images were analyzed using the Pixelink Microscopy-SW software (Pixelink).

### Statistical Analysis

For the qRT-PCR and phenotypic analyses, the data were recorded as the mean ± SEs of a minimum of three independent biological replicates. The statistical differences between samples were analyzed by Student’s *t*-test and *p* ≤ 0.05 was considered as significant.

## Results

### Genome-Wide Identification of Carrot E2F/DP Sequences

**Figure 1:**
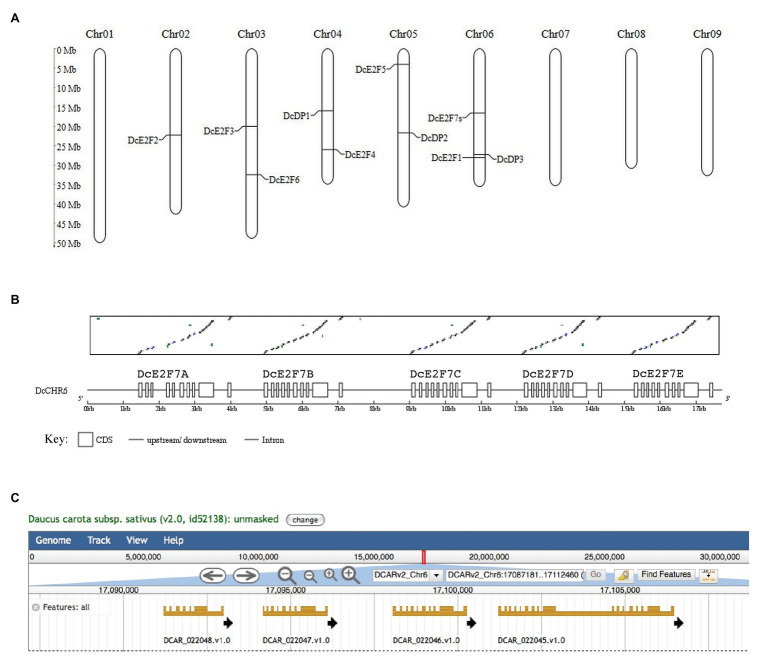
Chromosome map of the members of the E2F/DP family of *Daucus carota* and organization of the DcE2F7 locus. **(A)** Chromosome location of the E2F and DP genes on the nine chromosomes of carrot. **(B)** Matrix comparison between the *DcE2F7/DEL3* cDNA and the genomic region on chromosome 6 showing the five direct repeats of the *DcE2F7/DEL3* sequence, indicated below with letters A–E and displayed as boxes separated by single lines representing putative introns and intergenic regions. **(C)** Organization of the four predicted gene models at the *DcE2F7* locus as displayed by the genome browser available on the Phytozome platform (https://phytozome-next.jgi.doe.gov/info/Dcarota_v2_0).

Analyses of the *D. carota* subsp. sativus genomes of the Chinese variety Kuroda (Xu et al., 2014) and of the doubled-haploid, Nantes-type carrot (DH1; Iorizzo et al., 2016) allowed the identification of all the carrot E2F and DP genes. These analyses enabled the localization on chromosome 6 of *DcE2F1*, the E2F previously described (Albani et al., 2000), and revealed the presence in the carrot genome of three additional typical E2Fs, named *DcE2F2*, *DE2F3*, and *DcE2F4* based on the chromosomes on which they are located (Figure 1A). Searches for the atypical E2F/DEL sequences revealed the presence of seven homologous sequences in the carrot genome. *DcE2F5/DEL1* is located on chromosome 5, whereas the gene that we named *DcE2F6/DEL2* is on chromosome 3. The remaining atypical E2F/DEL sequences are tandemly repeated copies of the gene that we named *DcE2F7/DEL3* and are located on chromosome 6 (Figure 1B). The remarkable features of this locus are also shown in the genome browser displayed on the Phytozome platform,^14^ which however, predicts the presence of only four genes because parts of the last two repeated sequences are grouped together into a single gene model (Figure 1C). The BLAST analysis also revealed the presence of three DP genes, with *DcDP1* on chromosome 4, *DcDP2* on chromosome 5, and *DcDP3* on chromosome 6 (Figure 1A).

**Figure 2:**
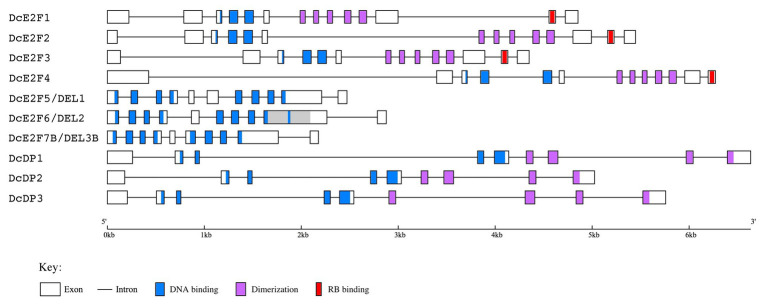
Gene structure of the carrot E2F/DP genes deduced from the cDNA sequences isolated. The coding exons are depicted as boxes while the introns are single lines. The conserved DNA binding, dimerization and RB binding regions are indicated with light blue, light purple and red colors, respectively, as described in the legend below. The repeated region in the ninth exon of *DcE2F6/DEL2* is shown in gray color.

To confirm the predicted gene models, the corresponding cDNAs were isolated by RT-PCR using mRNA extracted from carrot leaf and cell suspension cultures, and the resulting fragments were sequenced to verify the match with the genomic sequences. cDNA sequences were obtained for all the additional unique E2F and DP genes and for a DcE2F7/DEL3 gene that is nearly identical to the predicted *DcE2F7B* gene model (Figure 1B). It is unclear whether the other DcE2F7-like gene models are functional genes but the *DcE2F7A* and *DcE2F7E* sequences contain termination codons in the predicted coding regions. This could also explain why parts of the *DcE2F7D* and *DcE2F7E* sequences are grouped in a single gene model in the genome browser displayed on the Phytozome platform (Figure 1C). The structure of the confirmed E2F and DP genes is shown in Figure 2 and their genomic coordinates, along with their genomic annotation on the Phytozome platform and the gene IDs in the NCBI database,^15^ are reported in Supplementary Table S3. The correspondence between the E2F and DP cDNA sequences obtained and the genomic sequences of the DH1 and Kuroda varieties described on the Phytozome platform and on the CarrotDB database is reported in Supplementary Table S4.

The exon organization of the four typical E2Fs appears conserved and suggests that all of them could originate from a single ancestral DcE2F gene. A conserved exon organization is also seen for the three DcDP genes, whereas some differences are found among the three atypical E2F/DEL genes, with the presence of an additional internal exon for *DcE2F5/DEL1* and the duplication of a portion of the ninth exon of *DcE2F6/DEL2* (Figure 2).

**Figure 3:**
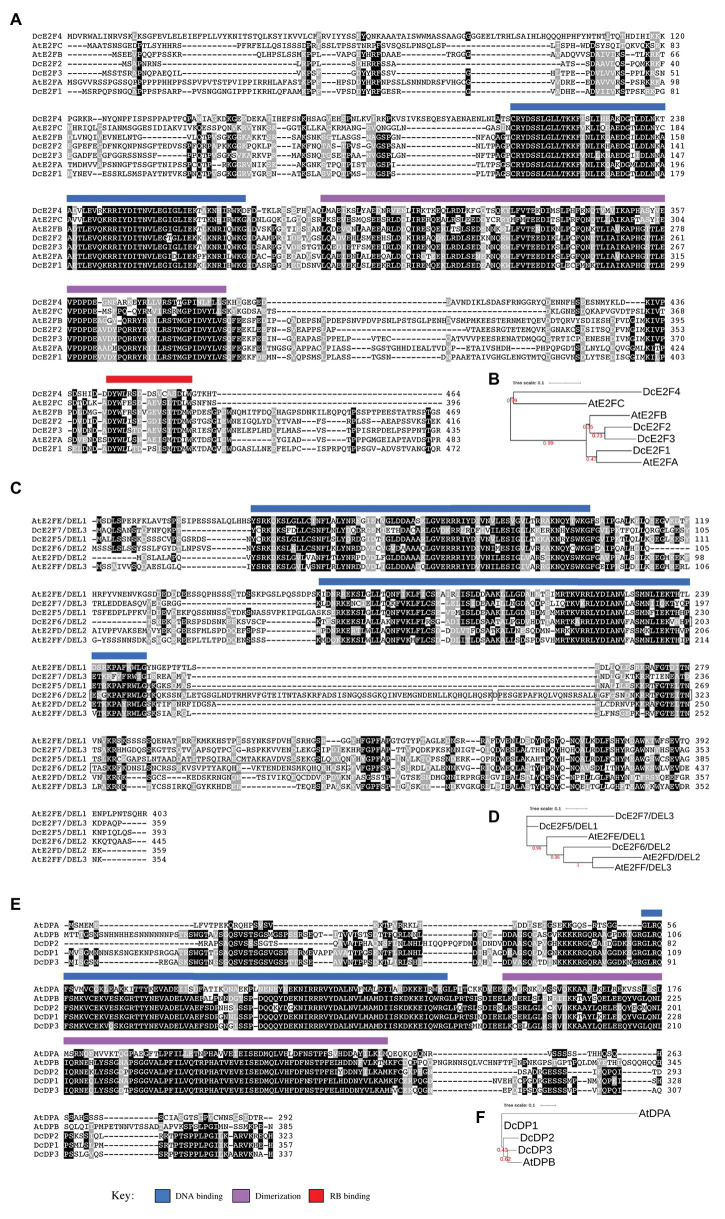
Sequence comparison of the carrot and Arabidopsis E2F and DP proteins. **(A,B)** Show, respectively, the amino acid sequence alignments and the phylogenetic tree of the carrot and Arabidopsis typical E2Fs. **(C,D)** Describe the same for the atypical E2Fs whereas **(E,F)** show the comparison of the DP proteins. The conserved DNA binding, dimerization, and RB binding regions are indicated by light blue, light purple, and red bars, respectively, as described in the key below. The repeated region in DcE2F6/DEL2 is boxed. The phylogenetic trees were constructed using the maximum-likelihood method implemented in the PhyML program (v3.1). Numbers shown in red at branch nodes indicate bootstrap values.

The typical DcE2F cDNAs encode proteins ranging in size from 416 (DcE2F2) to 472 (DcE2F1) amino acids that possess conserved DNA-binding, dimerization, and RBR-binding domains (Figure 3A). Compared to the previously published *DcE2F1* cDNA sequence, the *DcE2F1* genomic sequence reveals an amino terminal extension of the coding region linked to the presence of an in-frame ATG triplet located 123 bp upstream to the previous start codon that increases by 41 aa, the size of the DcE2F1 protein. Sequence comparisons and maximum-likelihood phylogenetic analyses of the carrot and Arabidopsis proteins using the PhyML program reveal higher homology of DcE2F1 to AtE2FA, whereas DcE2F4 appears to be more homologous to AtE2FC. DcE2F2 and DcE2F3 are instead grouped at a separate node together with AtE2FB (Figure 3B) although it is not clear whether any of them could be an ortholog of AtE2FB in view of the relatively low bootstrap value. To confirm and extend these results, phylogenetic analyses were also conducted including the E2F and DP sequences identified in the genome of coriander (Song et al., 2020), a closely related species that together with carrot belongs to the Apiaceae family. The FastTree phylogenetic analysis that describes the relationship of the carrot, coriander, and Arabidopsis E2F/DP proteins identified the coriander orthologs of the carrot E2F and DP proteins and confirmed the predicted orthology of DcE2F1 to AtE2FA and DcE2F4 to AtE2FC, whereas the orthology of DcE2F2 or DcE2F3 to AtE2FB remained unclear (Supplementary Figure S1). The orthology of DcE2F1 to AtE2FA is also supported by the high level of amino acid identity between these two proteins (Supplementary Table S5A). In contrast, the level of amino acid identity between DcE2F4 and AtE2FC is not as high although these E2Fs, as the coriander protein encoded by the *Cs10G01453* gene, show truncated carboxy-termini which end shortly after the RBR-binding domain (Figure 3A). Analysis of gene synteny of the carrot and Arabidopsis E2F loci confirmed the orthology between *DcE2F1* and *AtE2FA* but not between the other carrot and Arabidopsis E2Fs whose genomic regions are not clearly syntenic (Supplementary Figure S2A).

The atypical DcE2F cDNAs encode proteins that possess the two conserved DNA-binding domains and range in size from 359 (DcE2F7/DEL3) to 445 (DcE2F6/DEL2) amino acids (Figure 3C). The larger size of DcE2F6/DEL2 is linked to the duplication of a portion of 81 aa, boxed in Figure 3C, which includes the end of the second DNA-binding domain. The same duplication is found also in the atypical E2F encoded by the *Cs01G02190* gene of coriander (Supplementary Figure S1) and could be a specific feature of these E2F/DEL genes in the Apiaceae family because it is not found in the atypical E2Fs of other plant species so far reported. According to the maximum-likelihood phylogenetic analyses, DcE2F6/DEL2 clusters together with AtE2FD/DEL2 and AtE2FF/DEL3, whereas DcE2F5/DEL1 and DcE2F7/DEL3 are placed in separate branches and a clear orthology between the carrot and Arabidopsis atypical E2Fs could not be inferred (Figure 3D; Supplementary Figure S1). However, the analysis of gene synteny of the carrot and Arabidopsis E2F/DEL loci supports the orthology of *DcE2F6/DEL2* to *AtE2FD/DEL2* (Supplementary Figure S2B), although the alignment of the carrot and Arabidopsis atypical E2F protein sequences reveals higher amino acid identity of DcE2F6/DEL2 to AtE2FF/DEL3 and AtE2FE/DEL1 than to AtE2FD/DEL2 (Supplementary Table S5B).

The DcDP proteins possess conserved DNA-binding and dimerization domains and their size ranges from 323 (DcDP2) to 357 (DcDP1) amino acids (Figure 3E). The maximum-likelihood phylogenetic analyses reveal that DcDP2 and DcDP3 cluster together with the Arabidopsis AtDPB (Figure 3F; Supplementary Figure S1) and the analysis of gene synteny of the carrot and Arabidopsis DP loci supports the orthology of *DcDP2* to *AtDPB* (Supplementary Figure S2C). Moreover, all the carrot DP proteins show higher amino acid identity to AtDPB than to AtDPA of Arabidopsis (Supplementary Table S5C).

### Expression Analysis of the Carrot E2F and DP Genes

**Figure 4:**
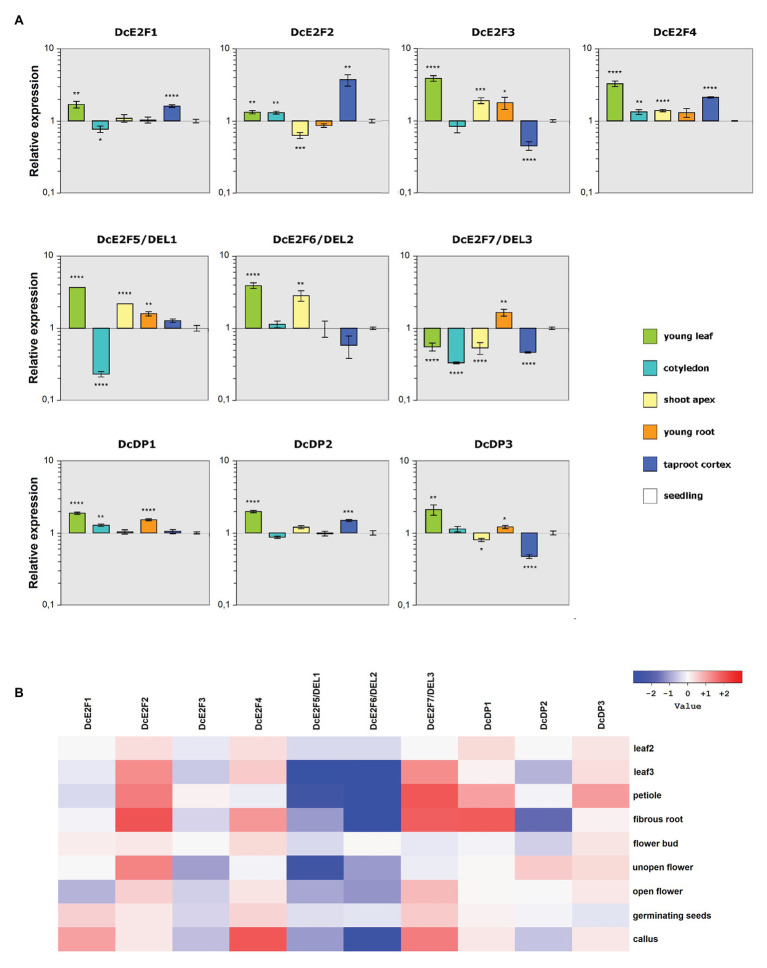
Patterns of expression of the carrot E2F and DP genes. **(A)** Quantitative reverse transcription PCR (qRT-PCR) analysis of the relative expression levels of the *DcE2F* and *DcDP* genes in representative organs compared to whole seedlings. The qRT-PCR analyses were repeated three times using independentbiological replicates and quantification was normalized to carrot actin RNA levels. Mean values are reported and the bars show SEs. Statistically significant difference in relative values of expression compared with whole seedlings was determined by Student’s *t*-test; ^*^p < 0.05, ^**^p < 0.01, ^***^p < 0.001, ^****^p < 0.0001. **(B)** Gene expression analysis based on the RNA-seq data. Differential gene expression ratios in various organs vs. stage 1 leaves (0.5–1 cm sprouts), computed with DESeq2. The ratios are expressed as Log2 values and displayed as a heatmap. Leaf 2 represents 2–2.5 cm long leaves, non-expanded, whereas Leaf 3 represents expanded 7–8 cm long leaves.

To analyze the expression of the carrot E2F/DP genes in different organs and in parts of the plant associated with cell proliferation, qPCR analyses were performed on reverse transcribed RNA isolated from 1-week-old seedlings or from leaves, cotyledons, shoot apices, and roots of 2-week-old plants, as well as from the cortex of mature taproots (Figure 4A). Additional expression analyses were carried out *in silico*, examining RNA-Seq data available for various *D. carota* organs which are reported in the NCBI Sequence Read Archive database (Supplementary Table S2). Although these SRA libraries represent data obtained from single samples and should be examined cautiously, their analysis can be useful to confirm and expand the results of the qRT-PCR analysis. Transcripts per million (TPM) values were calculated with Salmon and differential expression analyses were performed with the DESeq2 package (Figure 4B). The TPM values obtained from these analyses revealed a particularly strong expression of *DcE2F2*, *DcDP1*, and *DcDP3* (Supplementary Figure S3), but extremely low expression of the *DcE2F7/DEL3* gene, which showed no reads for three of the RNA-Seq samples (Supplementary Table S6), whereas variable levels of expression were calculated for the remaining E2F/DP genes. Moreover, all the carrot E2F and DP genes appeared to be differentially expressed in some of the organs (Supplementary Figure S4).

According to the qRT-PCR analyses (Figure 4A), the typical *DcE2F2* gene showed its maximal expression in the mature taproot cortex and its lowest expression in the shoot apices, with lower expression also in young roots compared to seedlings. The remaining typical DcE2Fs were instead all strongly expressed in young leaves and were also well expressed in shoot apices and young roots. *DcE2F1* and *DcE2F4* were highly expressed in the taproot cortex, whereas *DcE2F3* shows its lowest level of expression in this tissue. The differential expression analyses derived from the RNA-Seq data confirm the distinctive patterns of expression of the carrot E2F/DP genes (Figure 4B), with decreasing expression of *DcE2F1* and *DcE2F3* in older leaves compared to young leaves. Conversely, both *DcE2F2* and *DcE2F4* are more expressed in older leaves than in young leaves and show high expression also in fibrous roots. High expression of *DcE2F2* is found also in unopen flowers, in which low expression of *DcE2F3* is seen instead. Overall, according to both the qPCR and RNA-Seq analyses, the *DcE2F3* gene appears predominantly expressed in organs in which cell proliferation occurs, whereas its expression in organs with differentiated and undividing cells is considerably lower. Also *DcE2F1* and *DcE2F4* are well expressed in young leaves and in callus, both containing proliferating cells, but their expression is high also in some differentiated tissues, as revealed by the strong expression in the taproot cortex (Figure 4A). Conversely, the expression of *DcE2F2* is generally low in organs characterized by cell proliferation but is consistently higher in organs containing fully differentiated and non-dividing cells.

Concerning the atypical E2Fs, the qRT-PCR analysis revealed that both *DcE2F5/DEL1* and *DcE2F6/DEL2* are strongly expressed in young leaves and are also highly expressed in the shoot apices (Figure 4A). Conversely, the *DcE2F7/DEL3* gene is maximally expressed in young roots but shows low expression in young leaves and in shoot apices, as well as in the cotyledons and in the taproot cortex. Moreover, according to the SRA analysis, both *DcE2F5/DEL1* and *DcE2F6/DEL2* are less expressed in older leaves and in petioles than in young leaves, whereas *DcE2F7/DEL3* shows the opposite pattern and is expressed more in mature leaves and in petioles than in young leaves (Figure 4B). These results suggest a preferential expression of the atypical *DcE2F5/DEL1* and *DcE2F6/DEL2* genes in organs undergoing cell proliferation, whereas *DcE2F7/DEL3* is most expressed in mature leaves, although its expression is also high in young roots.

Distinctive spatial patterns of expression are also seen for the DcDP genes and, although all three genes are strongly expressed in young leaves (Figure 4A), *DcDP1* shows also high expression in young roots, whereas *DcDP2* is highly expressed in the taproot cortex, in which *DcDP3* shows the lowest expression instead. Moreover, the expression of *DcDP2*, but not of the other DcDPs, appears to decrease significantly in older leaves. *DcDP2* expression is also low in fibrous roots, in which high expression of *DcDP1* is seen (Figure 4B). It appears, therefore, that the expression of *DcDP2* is generally lower in organs containing non-dividing cells, whereas this is not the case for the other DcDP genes.

In summary, in line with data concerning Arabidopsis and animal E2Fs, most of the carrot E2F and DP genes show distinctive profiles of expression that suggest different roles of the various members of the E2F/DP family in carrot. In particular, the typical *DcE2F1*, *DcE2F3*, and *DcE2F4*, along with the atypical E2Fs *DcE2F5/DEL1* and *DcE2F6/DEL2* genes, are highly expressed in organs with dividing cells and are likely to be involved in the control of cell proliferation in carrot plants.

### *In silico* Analysis of the DcE2F and DcDP Promoters Reveals Common and Distinctive Features Suggesting Interplays Among the Carrot E2F Genes

Although post-transcriptional control of RNA stability can also affect transcript levels, the differential expression of genes is strongly correlated to the activity of their promoters. The transcriptional regulation of plant and animal E2F genes has been investigated in a limited number of studies and transcriptional interplays are known to occur among the E2F genes, many of which appear to be E2F-regulated (Vandepoele et al., 2005; Sozzani et al., 2006, 2010; Berckmans et al., 2011a; Kent and Leone, 2019). However, also other transcription factors have been reported to be involved in the control of some of the E2Fs. In Arabidopsis, the lateral organ boundary proteins LBD18 and LBD16 have been reported to regulate AtE2FA (Berckmans et al., 2011b; Ikeuchi et al., 2018) and Myb-like and PIF4 factors could also be involved in the control of some of the E2Fs (Berckmans et al., 2011b; Oh et al., 2012). In animal cells, Myc-mediated positive regulation of the *E2F1*, *E2F2*, and *E2F3a* genes has been described (Sears et al., 1997; Adams et al., 2000) but also YB-1, EKLF/KLF1, and NRF-1/α-PAL factors, have been shown to control some of the animal E2Fs (Efiok and Safer, 2000; Kherrouche et al., 2004; Tallack et al., 2009; Lasham et al., 2012; Ilsley et al., 2017). Although plant homologues of NRF-1/α-PAL and EKLF are not known, Myc proteins as well as CSP factors, related to YB-1, are found in plants.

**Figure 5:**
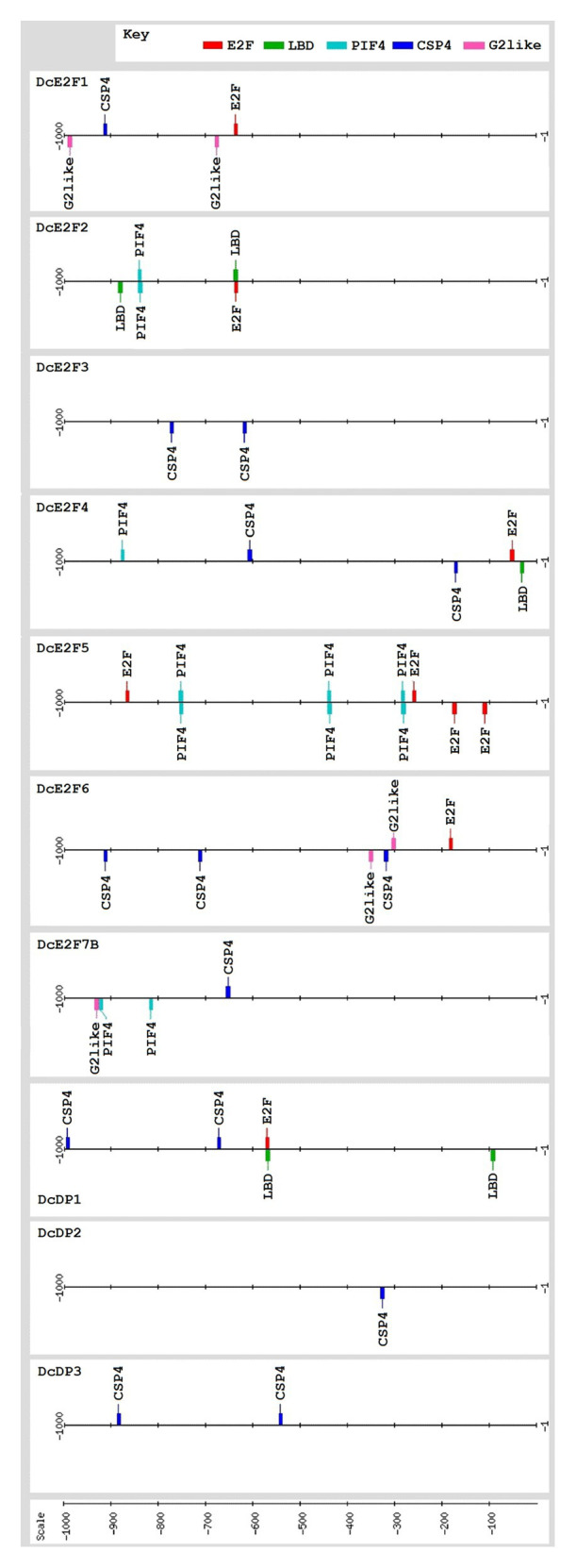
The promoters of the carrot E2F and DP genes contain common and distinctive *cis*-elements and several of them are likely to be E2F-regulated and/or controlled by CSP factors. Map of the relevant *cis*-acting elements identified in the 1,000 bp 5'flanking region upstream of the presumed ATG start codon of the E2F and DP genes. The relevant regulatory elements include the consensus binding sites for E2F (NNTSSCGSN), LBD (NNGCGGCWN), CSP4 (KTTTTWTTN), and PIF4 (NCACRTGNN), which is bound also by Myc proteins, and the G2-like site (NRGAATMTN) which is recognized by Myb factors. The *cis*-elements are displayed with different colors as indicated in the key. The map was created using the drawing tool of the RSAT (Regulatory Sequence Analysis Tools (RSAT) platform (http://www.rsat.eu/).

To verify whether the expression of some of the carrot E2F and DP genes could be controlled by E2F activities or by relevant transcription factors described in other studies, the 5' flanking region of the 10 members of the carrot E2F/DP family, extending up to 1,000 bp upstream of the ATG codon, was analyzed for the presence of E2F *cis*-elements and of binding sites recognized by LBDs, Myb factors, and CSP proteins, as well as binding sites of PIF4 which are recognized also by Myc proteins (Figure 5). The results of these analyses revealed the presence of E2F binding sites in the 5' flanking region of six of the genes, with as many as four E2F *cis*-elements upstream of the coding region of *DcE2F5/DEL1* and single E2F sites in the 5' flanking regions of *DcE2F1*, *DcE2F2*, *DcE2F4*, *DcE2F6/DEL2*, and *DcDP1*. Conversely, E2F sites were not detected upstream of the coding region of *DcE2F3*, *DcE2F7/DEL3*, *DcDP2*, and *DcDP3*. Concerning other putative regulatory elements, the CSP4 binding site, which is recognized by plant homologues of the human YB-1 factor, was the most common *cis*-element overall and was found in one or more copies in the 5' flanking region of eight genes, with the exception of *DcE2F2* and *DcE2F5/DEL1*. Moreover, CSP4 sites, in one or two copies, were the only relevant regulatory elements identified upstream of *DcE2F3*, *DcDP2*, and *DcDP3* coding regions (Figure 5). PIF4 binding sites, which are bound also by Myc factors, were found in the 5' flanking region of *DcE2F2*, *DcE2F4*, *DcE2F5/DEL1*, and *DcE2F7/DEL3*, and three PIF4 sites with four E2F sites were the only relevant regulatory elements found upstream to *DcE2F5/DEL1*. LBD binding sites as well as the G2-like elements, recognized by Myb factors, were detected in the 5' flanking region of three of the genes, but these putative regulatory sites were never found together. G2-like elements were found in the 5' flanking region of *DcE2F1*, *DcE2F6/DEL2*, and *DcE2F7/DEL3* whereas LBD sites were identified upstream of the *DcE2F2*, *DcE2F4*, and *DcDP1* coding regions. Moreover, in both *DcE2F2* and *DcDP1* 5' flanking regions one of the two LBD sites overlapped the E2F element (Figure 5).

Overall, apart from the widespread occurrence of CSP4 binding sites, the distribution of the putative sites recognized by LBDs, PIF4, and Myb factors agrees with a differential regulation of the carrot E2F and DP genes, as highlighted also by their distinctive patterns of expression. Moreover, the presence of E2F binding sites in the 5' flanking region of six of the E2F/DP genes suggests that in carrot, as in Arabidopsis and in animals, extensive interplays among the E2F genes are likely to occur.

### The Ectopic Expression of *DcE2F1* Can Affect Embryo Development and Increases Cell Proliferation in Arabidopsis Seedlings

**Figure 6:**
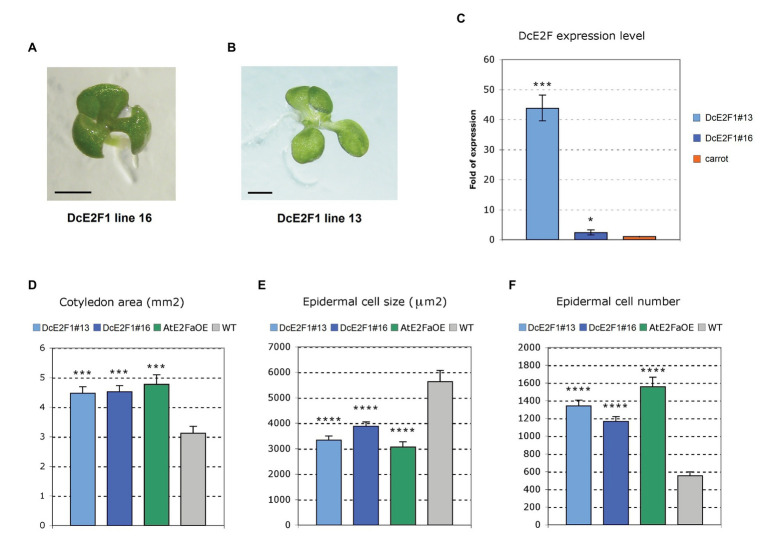
Ectopic expression of *DcE2F1* increases cell proliferation in transgenic seedlings. **(A,B)** Increased number of cotyledons in seedlings of transgenic Arabidopsis lines expressing ectopically *DcE2F1*. **(A)** Four-day-old tricotyledonous seedling of line DcE2F1#16 and **(B)** Six-day-old tetracotyledonous seedling of line DcE2F1#13. The presence of four cotyledons in **(B)** is associated with a duplication of the SAM. The length of the bars corresponds to 1 mm. **(C)** Expression of the *DcE2F1* mRNA in the transgenic Arabidopsis seedlings compared to the level of endogenous expression in carrot seedlings. The qRT-PCR analyses were repeated three times using independent biological replicates and quantification was normalized to 18S RNA levels. The mean values are reported and the bars show SEs. ^*^p < 0.05, ^***^p < 0.001 by Student’s *t*-test. **(D–F)** Phenotypic analysis of the cotyledons of seedlings expressing DcE2F1 or overexpressing AtE2FA compared to wild type controls. The phenotypic analysis was carried out on 12-day-old plants using 10–12 samples. The transgenic lines are characterized by larger cotyledons **(D)** that contain smaller cells **(E)** in higher number **(F)**. The size of the adaxial epidermal cells was calculated, counting the number of cells contained in an area of 100,000 μm^2^. The total epidermal cell number was estimated, dividing the cotyledon size by the cell size. The mean values are reported and the bars show SEs. Statistically significant difference in the transgenic lines compared with wt controls was determined by Student’s *t*-test; ^***^p < 0.001, ^****^p < 0.0001.

Sequence comparisons of the DcE2F and AtE2F proteins revealed higher homology of DcE2F1 to AtE2FA than to the other carrot or Arabidopsis E2Fs (Figure 3B and Supplementary Table S5A). Moreover, the transient expression of DcE2F1 together with a DP protein can strongly transactivate an E2F-responsive promoter in plant cells (Albani et al., 2000) and suggests that DcE2F1 is an activating E2F that, like Arabidopsis AtE2FA and AtE2FB, could promote plant cell proliferation. To investigate the role of DcE2F1 in plant growth, the effects of its ectopic expression were analyzed in transgenic Arabidopsis lines harboring a gene construct containing the previously described *DcE2F1* cDNA (Albani et al., 2000) placed under the control of the double 35S promoter. Most remarkably, the T2 progeny of eight of the 23 lines isolated revealed the occasional but consistent production of tricotyledonous seedlings (Figure 6A), a phenotype not observed in control untransformed seedlings. Although this phenotype was found at very low frequency (<1%), it was inherited with the same frequency in the progeny of the selfed T2 plants. Moreover, for line DcE2F1#13, a tetracotyledonous seedling with two shoot apical meristems was also observed in the T3 progeny of a tricotyledonous T2 plant (Figure 6B). Lines with single inserts were identified and two of them, DcE2F1#13 and DcE2F1#16, that are both characterized by the production of tricotyledonous seedlings, were analyzed by qRT-PCR to assess the level of expression of *DcE2F1*. In seedlings of both lines, the accumulation of *DcE2F1* transcripts is higher than the physiological level of expression measurable in carrot seedlings (Figure 6C). The expression driven by the double 35S promoter is particularly strong in line DcE2F1#13, showing the most striking phenotype, where *DcE2F1* expression is over 40 times higher than in carrot seedlings. In line DcE2F1#16, instead, the expression of *DcE2F1* is only about 2.3-fold higher than in the carrot seedlings.

**Figure 7:**
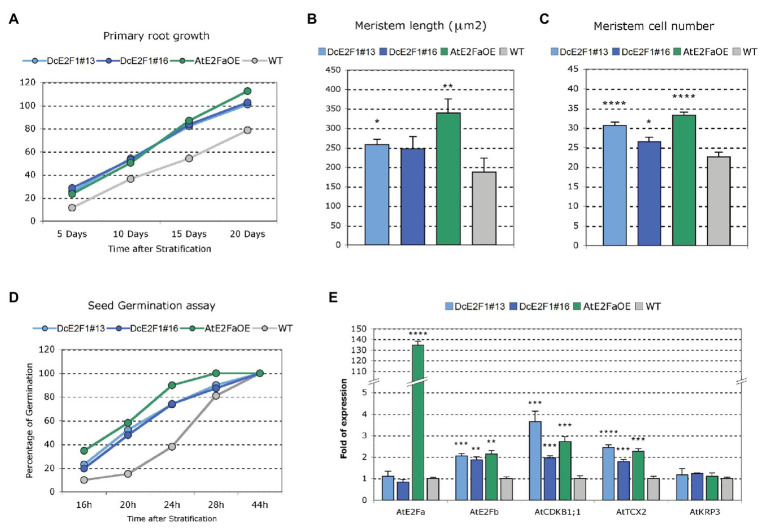
Ectopic expression of *DcE2F1* in transgenic plants affects root growth and seed germination, and upregulates *AtE2FB* and other E2F-responsive genes. **(A–C)** Phenotypic analysis of the roots of plants expressing DcE2F1 or overexpressing AtE2FA compared to wild type controls. **(A)** Primary root growth of Arabidopsis plants expressing *DcE2F1* or overexpressing *AtE2FA* compared to wild type plants. The mean primary root length was significantly longer for all transgenic genotypes compared with WT. Significance of the difference in length was determined by Student’s *t*-test; *n* = 30 (*p* ≤ 0.001). **(B)** Increased meristem length and **(C)** increased cell number in the RAM of seedlings expressing *DcE2F1* or overexpressing *AtE2FA* compared to wild type controls. The analysis was carried out on 5-day-old seedlings using 10–12 samples. Mean values are reported and the bars show standard errors. Statistically significant difference for the transgenic lines compared with wt controls was determined by Student’s *t*-test; ^*^p < 0.05, ^**^p < 0.01, ^****^p < 0.0001. **(D)** Seed germination assay showing an acceleration of germination in Arabidopsis lines expressing *DcE2F1* or overexpressing *AtE2FA* The analyses were carried out at 16, 20, 24, 28, and 44 h after stratification; *n* = 50. **(E)** Analysis of the expression of *AtE2FA*, *AtE2FB*, *AtCDKB1;1*, *AtTCX2*, and *AtKRP3* in seedlings expressing *DcE2F1* or overexpressing *AtE2FA* compared to wt seedlings. The qRT-PCR analyses were repeated three times using independent biological replicates and quantification was normalized to 18S RNA levels. Mean values are reported and the bars show SEs. Statistically significant difference in relative values of expression in the transgenic lines compared with wt controls was assessed by Student’s *t*-test; ^**^p < 0.01, ^***^p < 0.001, ^****^p < 0.0001.

Further analyses were performed to evaluate the effects of the ectopic expression of *DcE2F1* on cell proliferation. The same analyses were conducted on control untransformed plants and on a transgenic line overexpressing *AtE2FA* (AtE2FaOE), which also showed the occasional production of tricotyledonous seedlings with a similar low frequency as the DcE2F1 lines (SupplementaryFigure S5). Analysis of seedlings 12 days after seed stratification revealed a significant increase in the dimensions of the cotyledons in all the transgenic lines compared with wild type control (Figure 6D). Moreover, the size of the cotyledonary epidermal cells was significantly reduced and their number increased considerably (Figures 6E,F). In addition, the primary roots of the DcE2F1 lines and of the AtE2FaOE line appear to be significantly longer compared to control wild type plants (Figure 7A; Supplementary Figure S6). An analysis of the root apical meristems of young seedlings 5 days after seed stratification revealed an increase in the length of the meristematic zone (Figure 7B), which is associated with an increase in the number of cortical meristematic cells in all the transgenic lines (Figure 7C). However, the difference in root length compared to the wild type appears to be relatively constant during the growth of the transgenic plants and does not increase as the plants age (Figure 7A). This suggests that in the lines expressing *DcE2F1* or *AtE2FA* ectopically, the roots may have emerged earlier or grown faster soon after germination but their growth rate is unlikely to be altered in older plants. Indeed, germination assays revealed that the seeds of all the transgenic lines germinated earlier than the wild type seeds (Figure 7D). Thus, although the size of the adult transgenic plants is not different compared to untransformed control plants, the ectopic expression of *DcE2F1* in Arabidopsis plants is able to increase cell proliferation in the embryo and in the young seedlings and appears to be equivalent to the overexpression of *AtE2FA*.

According to studies on the typical E2Fs of Arabidopsis, AtE2FA bound by RBR has been proposed to control cell proliferation competence in meristems by repressing premature differentiation (Magyar et al., 2012). Moreover, AtE2FA is believed to activate directly the expression of *AtE2FB*, which in turn can upregulate G2/M phase genes to promote cell proliferation. Therefore, analyses were conducted on the transgenic lines to verify whether the ectopic expression of *DcE2F1* in Arabidopsis plants can upregulate *AtE2FB*. Indeed, the results of qRT-PCR analyses revealed an increased expression of *AtE2FB* in the DcE2F1 lines as well as in the AtE2FA overexpressing line (Figure 7E), whereas the expression of *AtE2FA* was not significantly affected by the ectopic expression of *DcE2F1* in Arabidopsis plants. Moreover, the DcE2F1 lines and the AtE2FaOE line showed upregulation of *AtCDKB1;1*, a known mitotic activator and a marker of cell proliferation. Increased expression was also seen for *AtTCX2*, a Tso1-like gene previously reported to be upregulated in *AtE2FA* overexpressing plants (Vandepoele et al., 2005), whereas the expression of the CDK inhibitor KIP-RELATED PROTEIN 3 (*AtKRP3*) was not affected, as also shown previously in AtE2FA overexpressing plants (Sozzani et al., 2006).

## Discussion

The discovery and preliminary characterization of the carrot DcE2F1 factor, one of the first plant E2Fs described, revealed a cell cycle-dependent pattern of expression and a strong transactivating potential that suggested an important role in the control of cell proliferation (Albani et al., 2000). With this report, we expanded the information on the carrot E2F factors, identifying all the members of the E2F/DP gene family and analyzing their patterns of expression in various carrot organs. Moreover, we investigated the role of DcE2F1 examining the effects of its ectopic expression in transgenic Arabidopsis plants, which indicate that, like the Arabidopsis AtE2FA factor, DcE2F1 can upregulate cell proliferation.

The search for E2F and DP sequences in the fully sequenced genome of *D. carota* and the isolation of the corresponding cDNA sequences revealed the presence of four typical E2F genes, three atypical E2F/DEL genes, and three DP members. However, it is noteworthy that the DcE2F7/DEL3 locus in chromosome 6 is characterized by the presence of a cluster of five tandemly repeated copies of the very similar genic sequence. We could isolate a cDNA clone corresponding to the member called *DcE2F7B/DEL3B* and is not clear whether other members of the cluster could also be functional genes. In this respect, stop codons in two of the DcE2F7/DEL3 genomic sequences appear to preclude the coding of full proteins but this does not exclude that other members of the cluster in addition to *DcE2F7B/DEL3B* could be expressed and represent additional components of the E2F/DP gene family of carrot.

The phylogenetic comparative analysis of the amino acid sequences of the carrot and Arabidopsis proteins suggests that *DcE2F1* is an ortholog of *AtE2FA*, as supported also by a partial synteny of their genomic loci, whereas *DcE2F2* and *DcE2F3* are more similar to *AtE2FB* and *DcE2F4* is more homologous to *AtE2FC*. Concerning the atypical E2F/DELs, *DcE2F6/DEL2* shows partial gene synteny with the *AtE2D/DEL2* and the DcE2F6/DEL2 protein is more similar to the Arabidopsis AtE2FD/DEL2 and AtE2FF/DEL3 factors than to the other carrot atypical E2Fs although, remarkably, it possesses a duplication of a portion of 81 aa at the end of the second DNA-binding domain of the protein. This feature is also seen in the ortholog from coriander, a species of the same family of carrot, but is not found in any of the atypical E2F/DELs so far described in other plant families. The duplication of domains in proteins is often associated with functional or structural features (Lees et al., 2016) but it is not clear whether the duplicated region in DcE2F6/DEL2 could reflect specific roles of this atypical E2F. With respect to the DcDPs, a partial gene synteny suggests a possible orthology between *DcDP2* and *AtDPB* but all the DcDP proteins are actually more similar to AtDPB and none of them is expected to be an ortholog of AtDPA.

The patterns of expression of the carrot E2F and DP genes in various organs revealed distinctive features, with maximal expression of *DcE2F1*, *DcE2F3*, and *DcE2F4* in organs containing proliferating cells, in which high expression was seen also for the atypical E2Fs *DcE2F5/DEL1* and *DcE2F6/DEL2*. On the contrary, the *DcE2F2* and *DcE2F7/DEL3* genes were generally more expressed in mature organs, where cells are not dividing. A particularly high expression of *DcE2F2* was seen in the cortex of mature taproots, where *DcE2F1*, *DcE2F4*, and *DcDP2* also show considerable levels of expression. Thus, the patterns of expression of *DcE2F3* and of both *DcE2F5/DEL1* and *DcE2F6/DEL2* suggest a strong involvement of these genes in cell proliferation, whereas *DcE2F1* and *DcE2F4* could act more broadly and could be involved in both cell proliferation and mature cell activities. *DcE2F2* and *DcE2F7/DEL3* are instead likely to regulate preferentially functions associated with differentiated cells. In this respect, new roles of the E2F factors are emerging in both plant and animal systems and can explain the different patterns of expression of the carrot E2F and DP genes. Most remarkably, in agreement with studies reported also in animal systems, there is evidence that cell proliferation in plants is not linked unconditionally to E2F-dependent transcriptional activation because Arabidopsis plants lacking the three typical E2Fs are sterile but appear to grow normally (Yao et al., 2018). These results revealed a crucial role of the activating E2Fs in plant germline development and the high expression of *DcE2F2* and *DcE2F4* in flower buds and open flowers could be associated to these functions. With respect to other roles, studies in Arabidopsis revealed that AtE2FC is involved in the regulation of secondary cell wall biosynthesis genes in a dose-dependent manner, because low doses of AtE2FC can act as a repressor together with RBR, whereas moderate levels of the factor can activate VND genes and promote secondary wall synthesis (Taylor-Teeples et al., 2015). Moreover, the typical AtE2Fs appear to be involved in plant effector-triggered immunity (Wang et al., 2014) because recognition of pathogen effectors by immune receptors leads to RBR hyperphosphorylation and over-activation of the typical E2Fs, which redundantly can trigger plant cell death and immunity. An involvement in plant immunity has been reported also for the atypical AtE2FE/DEL1 factor that, besides its role as repressor of the endocycle in mitotically active cells, has been shown to downregulate the expression of the EDS5 transporter of the hormone salicylic acid, a crucial regulator of plant defense response (Chandran et al., 2014). Thus, some of the carrot E2Fs are also likely to be involved in similar multiple processes.

The search of promoter *cis*-elements recognized by transcription factors shown to regulate E2F genes in plants or in animal cells revealed a widespread occurrence of CSP4 sites in the 5' flanking regions of most of the carrot E2F and DP genes, with the exception of *DcE2F2* and *DcE2F5/DEL1*. This *cis*-element is recognized by the CSP factors, which are plant homologues of the YB-1 factor that has been proposed to regulate several human E2F genes (Lasham et al., 2012). In Arabidopsis, AtCSPs are highly expressed in developing embryos and in shoot apices (Nakaminami et al., 2009) and have been shown to play important roles in plant development (Sasaki and Imai, 2012). However, the gene targets of plant CSPs are not known and their possible regulation of E2F genes needs to be assessed in future studies. Apart from the abundance of CSP4 sites, the distinctive distribution of putative sites recognized by LBDs, PIF4, and Myb factors indicates that distinct transcriptional regulatory mechanisms are likely to control expression of the carrot E2F and DP genes and could explain their differential patterns of expression. Moreover, E2F-binding sites, which are found in the 5' flanking regions of three of the typical E2Fs, two of the atypical E2F/DEL genes, and one of the carrot DPs, indicate that extensive interplays among the E2F genes are likely to occur, as already described in Arabidopsis and in animal cells (Vandepoele et al., 2005; Sozzani et al., 2006, 2010; Berckmans et al., 2011a; Kent and Leone, 2019). E2F sites are found in the 5' flanking regions of the atypical E2Fs *DcE2F5/DEL1* and *DcE2F6/DEL2*, which are highly expressed in organs containing proliferating cells, but are absent upstream to the coding region of *DcE2F7/DEL3*, whose expression is particularly high in mature organs. Moreover, *DcE2F3*, like *AtE2FA* in Arabidopsis, lacks E2F *cis*-elements in its 5' flanking region and thus is unlikely to be under the control of any of the other E2Fs, although its expression appears to be highly correlated with cell proliferation. Like *AtE2FA* in Arabidopsis, *DcE2F3* could be a regulator of other carrot E2Fs but, unlike *AtE2FA*, is not expected to be a target of LBD factors and the analysis of its 5' flanking region rather suggests a possible regulation by CSP proteins.

Sequence comparisons revealed that *DcE2F1* is likely the carrot ortholog of the *AtE2FA* gene of Arabidopsis. This evidence is supported also by the fact that the ectopic expression of *DcE2F1* in transgenic Arabidopsis plants gives rise to striking phenotypes that are very similar with those observed when overexpressing *AtE2FA*. In fact, the expression of *DcE2F1* driven by the constitutive 35S promoter leads to an upregulation of *AtE2FB* and promotes cell proliferation in young seedlings. Moreover, transgenic lines expressing *DcE2F1* ectopically or overexpressing *AtE2FA* yielded tricotyledonous seedlings with low frequency, a phenotype that was recently found in transgenic Arabidopsis lines overexpressing a truncated AtE2FB protein lacking the C-terminal RBR-binding domain (Őszi et al., 2020). The rare production of spontaneous tricotyledonous seedlings in untransformed Arabidopsis has been reported in some cases but in our experimental conditions and with several studies we could never observe tricotyledony in control Col-0 seedlings. Albeit with very low frequency (<1% of the seedlings), we were able to observe this phenotype in 35% of the transgenic DcE2F1 lines and in 20% of the AtE2FaOE lines isolated. This striking effect on embryo development is further highlighted by the identification of a T3 seedling of the highly expressing line DcE2F1#13 featuring four cotyledons and two SAMs. The low frequency of tricotyledony in the individual lines suggests that the effects of a misregulation of the E2F activities in developing embryos are largely counterbalanced by the strict regulatory pathways controlling embryo development and this could explain why this phenotype was not discovered previously in plants overexpressing the AtE2FA factor (De Veylder et al., 2002). During embryo development, cotyledon primordia initiate at positions of maximum auxin concentration resulting from polar PIN1 localization and various defects in cotyledon formation have been associated to altered auxin distribution or perception (Chandler, 2008). In Arabidopsis, auxin signaling has been linked to the upregulation of AtE2FA expression (Berckmans et al., 2011b) and to AtE2FB and AtE2FC protein turnover (Magyar et al., 2005; Jurado et al., 2010) but it is not known whether the activity of E2F factors can affect auxin metabolism or distribution. Many genetic pathways have been involved in cotyledon formation and in the *ALTERED MERISTEM PROGRAM1* mutant, an increase in the number of cotyledons has been linked to the expression of an AP2/ERF transcription factor that leads to enlarged shoot apical meristems and supernumerary stem cell pools (Yang et al., 2018). Thus, the observed occasional polycotyly in the DcE2F1 and AtE2FaOE transgenic lines could be linked to increased cell proliferation in the embryos or could indicate novel roles of these activating E2Fs in the control of gene pathways involved in cotyledon organogenesis.

Apart from the production of embryos with more than two cotyledons, the seedlings of the transgenic lines were characterized by a significant increase in the size of the cotyledons, whose cells were drastically reduced in size and more than doubled in number. A similar phenotype concerning cotyledon size and cell number has been reported also previously in Arabidopsis lines overexpressing *AtE2FA* or *AtE2FB* (De Veylder et al., 2002; Sozzani et al., 2006). Moreover, the transgenic DcE2F1 and AtE2FaOE lines are characterized by longer roots compared to untransformed plants and the analysis of their RAMs in very young seedlings revealed a significant increase in the meristem length and in the number of the meristem cortical cells. Interestingly, this root phenotype has not been reported previously in *AtE2FA* overexpressing plants and is very different from the phenotype shown by lines overexpressing *AtE2FB*, in which the cotyledons are also larger and contain more smaller cells but the roots are much shorter and are characterized by the presence of shorter isodiametric cells (Sozzani et al., 2006). The effects on root growth in the transgenic DcE2F1 and AtE2FaOE lines are likely to derive at least in part from an acceleration of radicle emergence. In fact, the seeds of these lines appear to germinate faster than the wild type seeds but the difference in length of the roots in the growing plants appears to be relatively constant and does not increase significantly in older plants. In this respect, although the seedlings of the transgenic plants possess larger cotyledons with more cells, their growth is normal and the size of the adult plants is not significantly different compared to the untransformed control plants. It appears, therefore, that the ectopic expression of *DcE2F1* or *AtE2FA* in the transgenic lines can significantly affect cell proliferation during the initial growth of the seedlings but is not likely to alter the proliferation of the meristematic cells once the correct hormonal balances are implemented to regulate plant development.

In summary, with this study, we described the E2F/DP gene family of *D. carota*, composed of four typical E2Fs, three atypical E2F/DEL members, and three DPs, and revealed an expansion of the DcE2F7/DEL3 locus that is characterized by the tandem duplication of five copies of the same genic sequence. Distinctive spatial patterns of expression confirmed an involvement of some of the DcE2Fs in the control of cell proliferation but also suggest unrelated roles for some of the family members. A clear involvement in cell proliferation is true for DcE2F1, the likely ortholog of the Arabidopsis AtE2FA factor, whose ectopic expression in Arabidopsis plants yields seedlings with increased cell number that can occasionally display polycotyly. These results expand our knowledge on the plant E2Fs and their involvement in cell proliferation and lay the ground for future investigations concerning the roles of the typical and atypical E2Fs in multiple processes in *D. carota*.

## Data Availability Statement

The datasets used in this study can be found in online repositories and their accession numbers can be found in the article/Supplementary Material.

## Author Contributions

DA designed this study, supervised the work, performed the bioinformatic analyses and wrote the manuscript with input from co-authors. LP, RG, and DF performed the experiments. LP, DF, HR, and DA analyzed the data. All authors contributed to the article and approved the submitted version.

### Conflict of Interest

The authors declare that the research was conducted in the absence of any commercial or financial relationships that could be construed as a potential conflict of interest.

## Footnotes

^1^http://apiaceae.njau.edu.cn:8080/carrotdb/

^2^https://phytozome-next.jgi.doe.gov/info/Dcarota_v2_0

^3^http://mg2c.iask.in/mg2c_v2.0/

^4^https://www.ebi.ac.uk/Tools/msa/muscle/

^5^https://embnet.vital-it.ch/software/BOX_form.html

^6^http://itol.embl.de/

^7^http://cgdb.bio2db.com/

^8^https://galaxy.pasteur.fr/

^9^http://itol.embl.de/

^10^http://primer3.ut.ee

^11^https://usegalaxy.org/

^12^http://www1.heatmapper.ca/

^13^http://www.rsat.eu/

^14^https://phytozome-next.jgi.doe.gov/info/Dcarota_v2_0

^15^https://www.ncbi.nlm.nih.gov/
