# Supplementary material for: Molecular Analysis of the E2F/DP Gene Family of Daucus carota and Involvement of the DcE2F1 Factor in Cell Proliferation
Source: Front Plant Sci. 2021 Mar 12;12:652570. doi: 10.3389/fpls.2021.652570 (PMC7994507; doi:10.3389/fpls.2021.652570)
Supplement: Supplementary file 1 [file Data_Sheet_1.docx]

**Supplementary data**


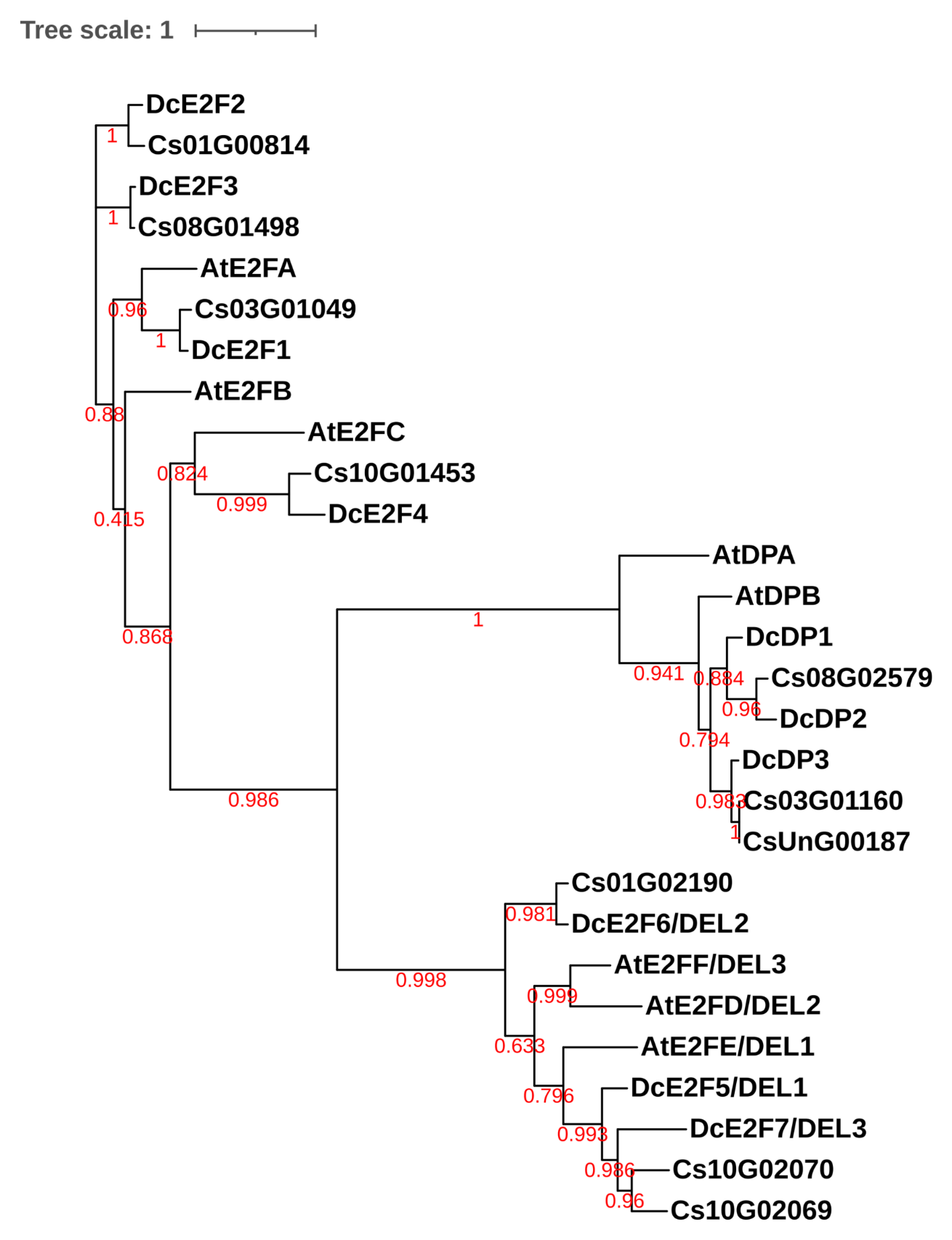


**Supplementary Figure S1.** Phylogenetic analysis of the relationship among carrot, coriander and Arabidopsis E2F and DP proteins. The phylogenetic tree was constructed using the FASTTREE program (v2.1.9) using LG substitution model and bootstrapping with 1000 replicates for branch support. The tree image was generated online using iTOL (<http://itol.embl.de/>). Numbers shown in red at branch nodes indicate bootstrap values.


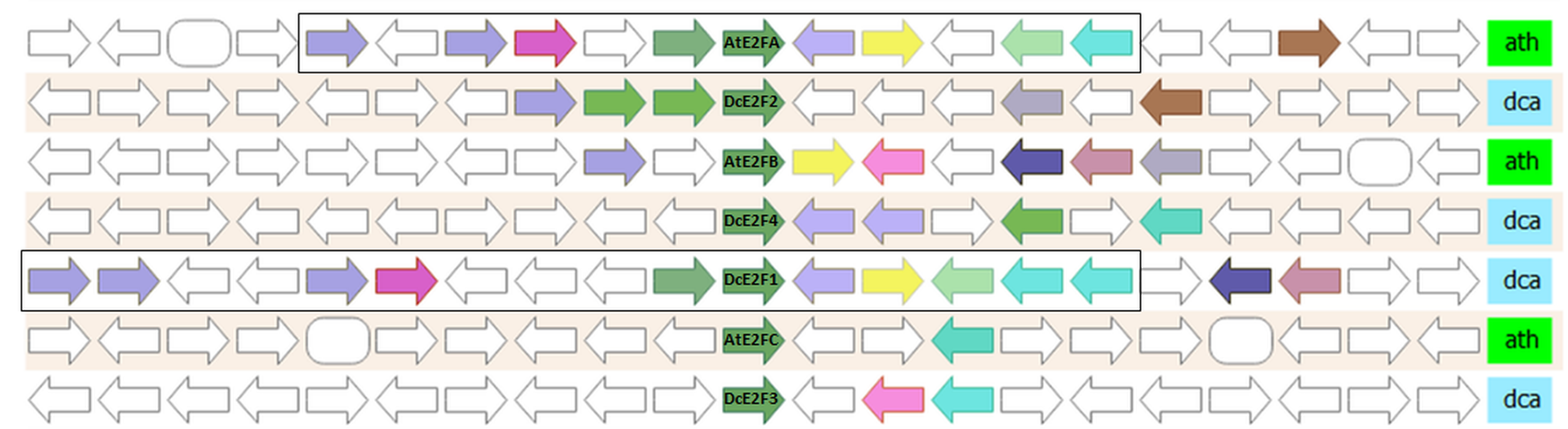

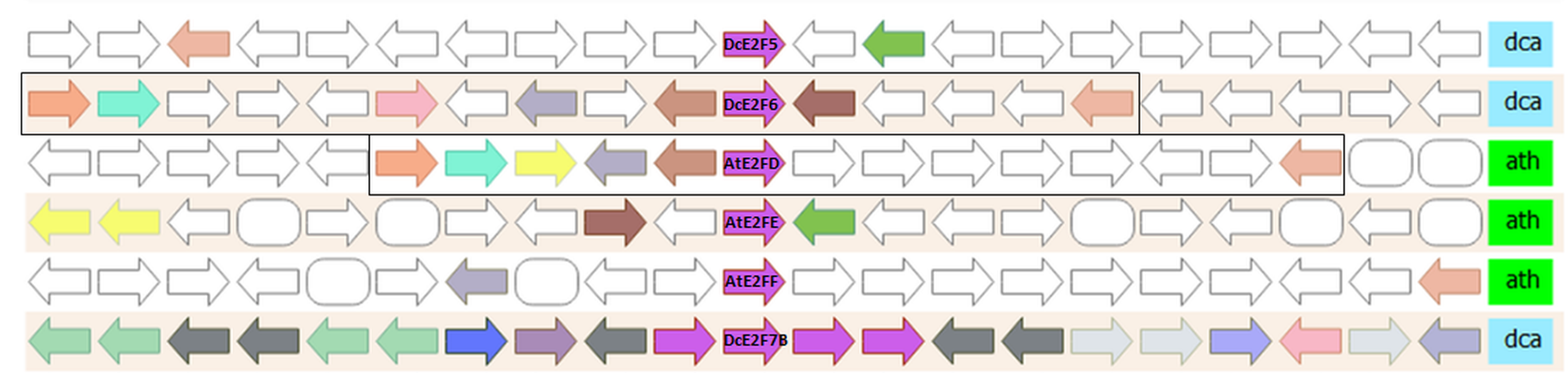

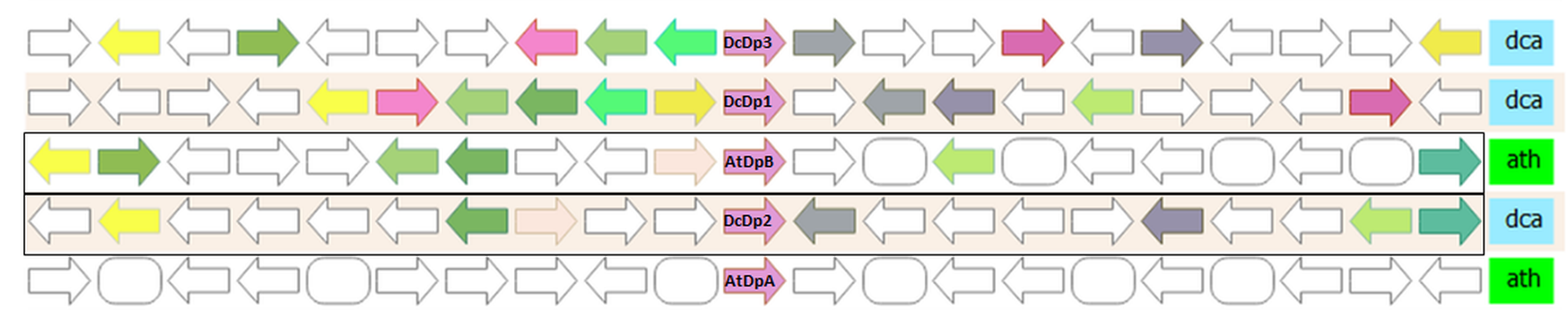


**A**

**B**

**C**

**Supplementary Figure S2.** Synteny maps of the genomic regions of carrot and Arabidopsis typical E2Fs (A), atypical E2F/DPs (B) and DP genes (C). The boxed regions highlight the synteny between the genomic regions of *DcE2F1* and *AtE2Fa*, *DcE2F6* and *AtE2FD*, as well as *DcDP2* and *AtDPB*. The maps were retrieved from the Dicots Plaza 4.0 Platform (<https://bioinformatics.psb.ugent.be/plaza/versions/plaza_v4_dicots/>).


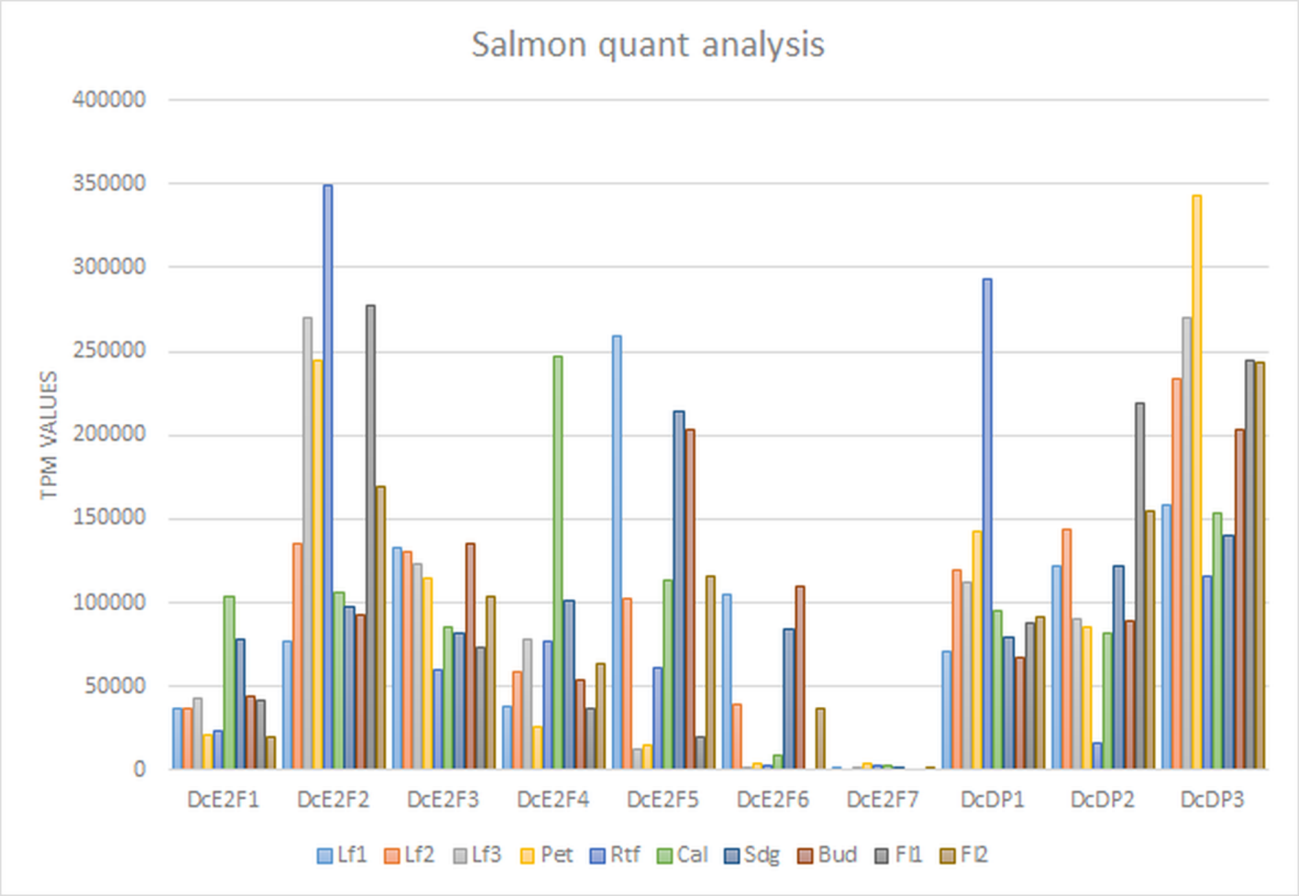


**Supplementary Figure S3.** Histogram showing the overall level of expression of each carrot E2F/DP gene based on the TPM values calculated on RNA Seq samples from 10 organs. The bars corresponding to each RNA Seq sample are shown in different colors. Lf1, stage 1 leaves (0.5-1 cm sprouts); Lf2, stage 2 leaves (2-2.5 cm leaves, non-expanded); Lf3, stage 3 leaves (expanded 7-8 cm leaves); Pet, petioles; Rtf, fibrous roots; Cal, callus; Sdg, germinating seeds; Bud, flower buds; Fl1, unopened flowers; Fl2, open flowers.


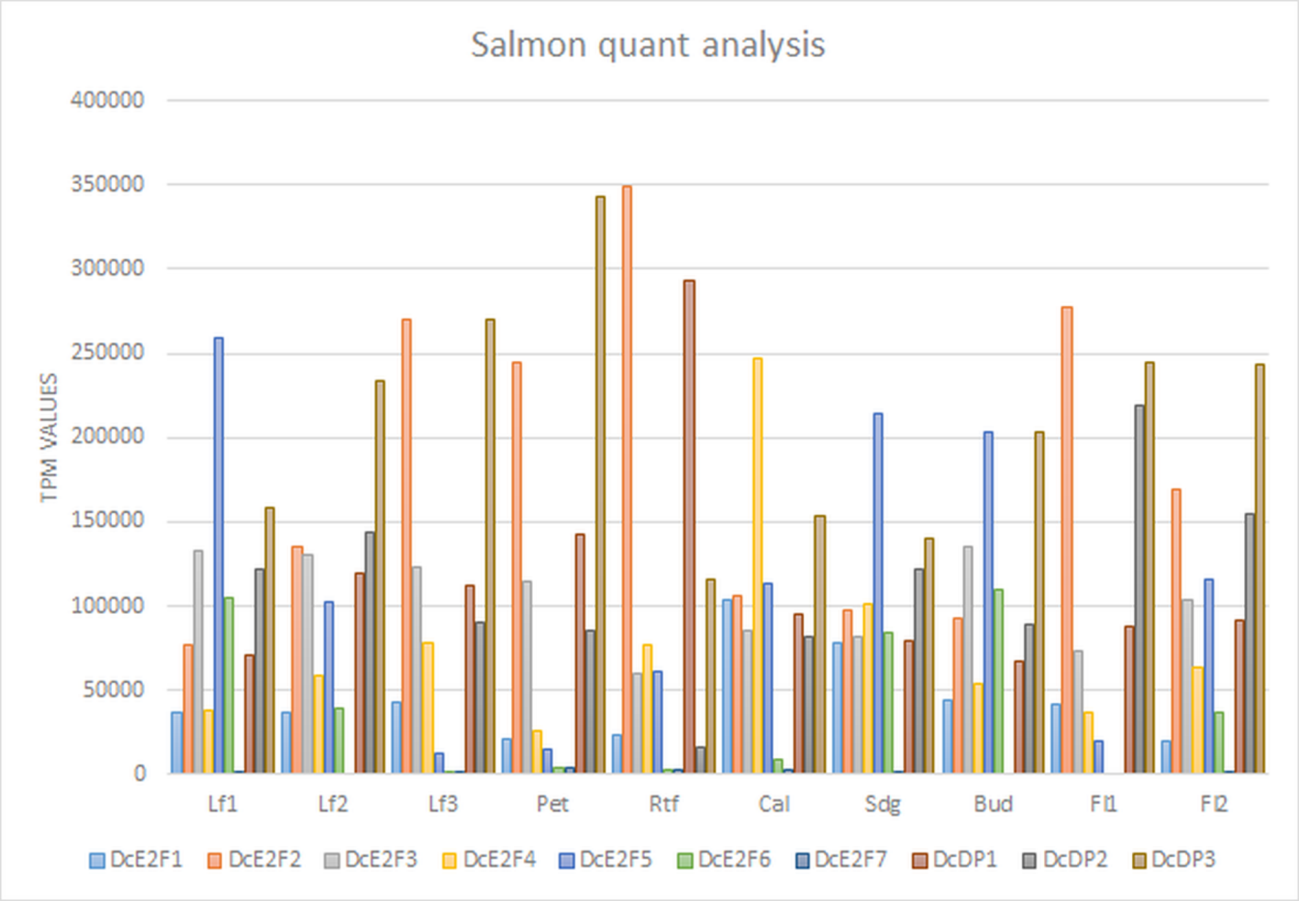


**Supplementary Figure S4.** Histogram showing the relative level of expression of the carrot E2F/DP genes in each of the 10 organs according to the TPM values calculated on the respective RNA Seq samples. The bars corresponding to each carrot E2F/DP gene are shown in different colors. . Lf1, stage 1 leaves (0.5-1 cm sprouts); Lf2, stage 2 leaves (2-2.5 cm leaves, not expanded); Lf3, stage 3 leaves (expanded 7-8 cm leaves); Pet, petioles; Rtf, fibrous roots; Cal, callus; Sdg, germinating seeds; Bud, flower buds; Fl1, unopen flowers; Fl2, open flowers.


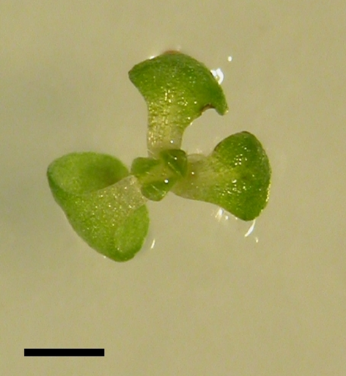


**Supplementary Figure S5.** Tricotyledonous seedling from the transgenic AtE2FaOE line. The bar length corresponds to 1 mm.

**
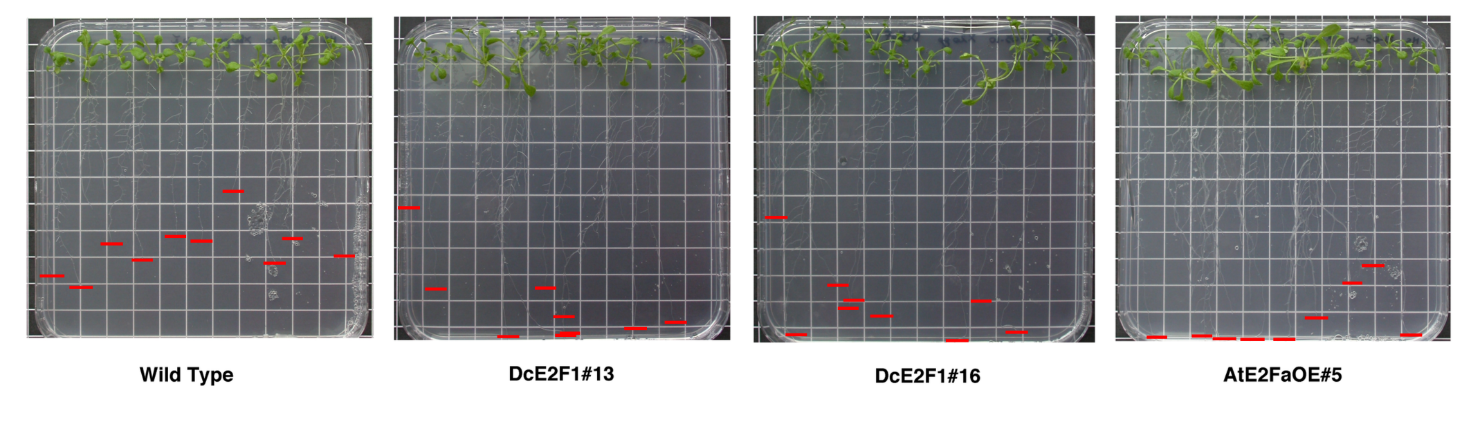
**

**Supplementary Figure S6.** Root length of plants of the two transgenic DcE2F1 lines of Arabidopsis and of the transgenic AtE2FaOE line compared to untransformed wild type plants. The position of the primary root tips is indicated with red bars.

**Supplementary Table S1.** Primers used for qRT-PCR analysis.

| Primer Name | Sequence |
| --- | --- |
| DcE2F1-F | TGTTTTGCAGGCAGAAGTTG |
| DcE2F1-R | GTGGTTCCATGTGGAGCTTT |
| DcE2F2-F | AAATGCAGGTGAAGGAAGGC |
| DcE2F2-R | TTCCAGACCCTGTACTCCAC |
| DcE2F3-F | AGTAGTTGTGCCCGAGGAAA |
| DcE2F3-R | TCGTTCCATTCAACCCCAGA |
| DcE2F4-F | GCCTGTATGCTGAAGACAACA |
| DcE2F4-R | AGCATGTGGAGCTTTTATCACC |
| DcE2F5-F | TGGACTTGATGATGCTGCTG |
| DcE2F5-R | TGCAGCGTTTTAGGAATGACT |
| DcE2F6-F | CAACAGCATTGCCAGGTGAT |
| DcE2F6-R | TCTGTTTCATCCCCAACCAC |
| DcE2F7-F | GCCTCAAGACAAACCCCAAG |
| DcE2F7-R | TCCCCGCAACTTCAGAATGA |
| DcDP1-F | CCTCATGCCACTGTAGAGGT |
| DcDP1-R | CTCTATCTCCAGGGCCATCG |
| DcDP2-F | GAAGGGTATATGATGCGCTG |
| DcDP2-R | CCTTCATACTGGTCTCTAGC |
| DcDP3-F | GGACTGCGAAGTAGGGTTGA |
| DcDP3-R | AGTCAAAATGCACCAGTTGC |
| DcACT-F | CTTGCTGGCCGTGATCTTAC |
| DcACT-R | CAGCTCCAATGGTGATAACT |
| AtE2Fa-F | TGATAGCCGTCAAAGCTCCT |
| AtE2Fa-R | TCGATGTCATGGTGTCCTGT |
| AtE2Fb-F | AAGCACCGAAAGAAACATGG |
| AtE2Fb-R | GTTTGTGGCTGCTCCAAGAT |
| AtCDKB1;1-F | CGATTACTCTGCGTCGAACA |
| AtCDKB1;1-R | TATGACAATGCGCAACACCT |
| AtTCX2-F | GGCGCTGCTTAACTTTTGAC |
| AtTCX2-R | TTTTTCGGCCACTTGAGACT |
| AtKRP3-F | TCTAGCACGAGGTCGATGTG |
| AtKRP3-R | TGACTTGCACCCATTCGTAA |
| 18S-F | CCTGCGGCTTAATTTGACTC |
| 18S-R | TTAGCAGGCTGAGGTCTCGT |

**Supplementary Table S2.** Accession number, size and sample information concerning the RNA-seq libraries used in this study.

| **Accession number** | **Sample name** | **Library size** | **Sample description** |
| --- | --- | --- | --- |
| [**SRX1136487**](https://www.ncbi.nlm.nih.gov/sra/SRX1136487%5baccn%5d)**:** | **Lf1** | 5.7G bases | leaves stage 1, 0.5-1 cm young sprout |
| [**SRX1136488**](https://www.ncbi.nlm.nih.gov/sra/SRX1136488%5baccn%5d)**:** | **Lf2** | 5.4G bases | leaves stage 2, 2-2.5 cm leaves, not expanded |
| [**SRX1136489**](https://www.ncbi.nlm.nih.gov/sra/SRX1136489%5baccn%5d)**:** | **Lf3** | 5.5G bases | leaves stage 3, 7-8 cm leaves, expanded |
| [**SRX1136490**](https://www.ncbi.nlm.nih.gov/sra/SRX1136490%5baccn%5d)**:** | **Pet** | 6.3G bases | 10 cm petiole from leaves stage3 |
| [**SRX1136494**](https://www.ncbi.nlm.nih.gov/sra/SRX1136494%5baccn%5d)**:** | **Rtf** | 4.8G bases | fibrous roots |
| [**SRX1136468**](https://www.ncbi.nlm.nih.gov/sra/SRX1136468%5baccn%5d)**:** | **Bud** | 5.9G bases | 0.5 mm buds |
| [**SRX1136470**](https://www.ncbi.nlm.nih.gov/sra/SRX1136470%5baccn%5d)**:** | **Fl1** | 4.2G bases | Whole flowers (not opened), 2 cm umbel |
| [**SRX1136486**](https://www.ncbi.nlm.nih.gov/sra/SRX1136486%5baccn%5d)**:** | **Fl2** | 8.9G bases | Whole flowers (opened), 2 cm umbel with flower at anthesis |
| [**SRX1136536**](https://www.ncbi.nlm.nih.gov/sra/SRX1136536%5baccn%5d)**:** | **Sd1** | 5.8G bases | Germinating seeds, at the beginning of germination |
| [**SRX1136495**](https://www.ncbi.nlm.nih.gov/sra/SRX1136495%5baccn%5d)**:** | **Cal** | 6.1G bases | callus |

**Supplemental Table S3.** Genomic coordinates of the validated coding sequences of the E2F and DP genes of *D. carota* along with their genomic annotation on the Phytozome platform and the gene IDs reported in the NCBI database (<https://www.ncbi.nlm.nih.gov/>).

| **Gene** | **Genomic coordinates** | **Gene Annotation** | **NCBI gene ID** |
| --- | --- | --- | --- |
| ***DcE2F1*** | Chr6:28916921-28921774 - strand | DCAR_020719 | LOC108192850 |
| ***DcE2F2*** | Chr2:2875053-22880499 - strand | DCAR_006178 | LOC108206188 |
| ***DcE2F3*** | Chr3:20587518-20591867 + strand | DCAR_010316 | LOC108211471 |
| ***DcE2F4*** | Chr4:26799543-26805813 + strand | DCAR_013863 | LOC108217718 |
| ***DcE2F5*** | Chr5:4176489-4178959 - strand | DCAR_016509 | LOC108222041 |
| ***DcE2F6*** | Chr3:33472799-33475675 - strand | DCAR_011188 | LOC108213233 |
| ***DcE2F7B*** | Chr6:17093947-17096123 + strand | DCAR_022047 | N/A # |
| ***DcDP1*** | Chr4:16456673-16463307 - strand | DCAR_014999 | LOC108216399 |
| ***DcDP2*** | Chr5:22307315-22312338 - strand | DCAR_017679 | LOC108220898 |
| ***DcDP3*** | Chr6:28051734-28057491 + strand | DCAR_020815 | LOC108227231 |

# Gene IDs at the NCBI database are reported for the following predicted members of the DcE2F7 gene cluster: *DcE2F7C*= LOC108226054, *DcE2F7D*=LOC108226055, *DcE2F7E*=LOC108226056.

**Supplementary Table S4.** Nucleotide differences of the coding sequences of the E2F and DP cDNAs of the cv. Lunga di Amsterdam compared to the exonic genomic sequences of the Kuroda and DH1 varieties. The differences are expressed as number of nucleotide changes/total nucleotides of the coding sequences.

|  | **Kuroda** | **DH1** |
| --- | --- | --- |
| ***DcE2F1*** | 8/1419 | 8/1419 |
| ***DcE2F2*** | 5/1251 | 22/1251 |
| ***DcE2F3*** | 1/1308 | 2/1308 |
| ***DcE2F4*** | 10/1395 | 1/1395 |
| ***DcE2F5*** | 0/1182 | 0/1182 |
| ***DcE2F6*** | 1/1338 | 16/1338 |
| ***DcE2F7B*** | 16/1080 | 16/1080 |
| ***DcDP1*** | 5/1074 | 6/1074 |
| ***DcDP2*** | 0/972 | 0/972 |
| ***DcDP3*** | 8/1014 | 1/1014 |

**Supplementary Tables S5.** Percent Identity Matrices of carrot and Arabidopsis E2F and DP proteins. The Identity Matrices were created aligning the amino acid sequences using MUSCLE (<https://www.ebi.ac.uk/Tools/msa/muscle/>).

**Table S5A. Typical E2F proteins Identity Matrix**

|  | DcE2F1 | DcE2F2 | DcE2F3 | DcE2F4 | AtE2FA | AtE2FB | AtE2FC |
| --- | --- | --- | --- | --- | --- | --- | --- |
| DcE2F1 | 100.00 | 56.68 | 55.31 | 39.29 | 62.65 | 52.27 | 42.94 |
| DcE2F2 | 56.68 | 100.00 | 66.34 | 37.22 | 56.42 | 55.45 | 41.43 |
| DcE2F3 | 55.31 | 66.34 | 100.00 | 36.22 | 53.14 | 55.24 | 42.82 |
| DcE2F4 | 39.29 | 37.22 | 36.22 | 100.00 | 36.43 | 37.01 | 36.95 |
| AtE2FA | 62.65 | 56.42 | 53.14 | 36.43 | 100.00 | 49.20 | 40.05 |
| AtE2FB | 52.27 | 55.45 | 55.24 | 37.01 | 49.20 | 100.00 | 41.00 |
| AtE2FC | 42.94 | 41.43 | 42.82 | 36.95 | 40.05 | 41.00 | 100.00 |

**Table S5B. Atypical E2F/DEL proteins Identity Matrix**

|  | DcE2F5 | DcE2F6 | DcE2F7B | AtE2FD | AtE2FE | AtE2FF |
| --- | --- | --- | --- | --- | --- | --- |
| DcE2F5/DEL1 | 100.00 | 46.13 | 53.50 | 42.33 | 49.87 | 46.59 |
| DcE2F6/DEL2 | 46.13 | 100.00 | 40.06 | 44.97 | 47.22 | 51.50 |
| DcE2F7B/DEL3B | 53.50 | 40.06 | 100.00 | 34.03 | 37.96 | 37.61 |
| AtE2FD/DEL2 | 42.33 | 44.97 | 34.03 | 100.00 | 43.84 | 59.82 |
| AtE2FE/DEL1 | 49.87 | 47.22 | 37.96 | 43.84 | 100.00 | 45.98 |
| AtE2FF/DEL3 | 46.59 | 51.50 | 37.61 | 59.82 | 45.98 | 100.00 |

**Table S5C. DP proteins Identity Matrix**

|  | DcDP1 | DcDP2 | DcDP3 | AtDPA | AtDPB |
| --- | --- | --- | --- | --- | --- |
| DcDP1 | 100.00 | 77.88 | 80.65 | 45.86 | 69.12 |
| DcDP2 | 77.88 | 100.00 | 74.19 | 41.79 | 65.81 |
| DcDP3 | 80.65 | 74.19 | 100.00 | 44.83 | 68.45 |
| AtDPA | 45.86 | 41.79 | 44.83 | 100.00 | 41.38 |
| AtDPB | 69.12 | 65.81 | 68.45 | 41.38 | 100.00 |

**Supplementary Table S6.** TPM Values from Salmon quant analysis on RNA Seq samples from 10 organs. Leaf1, stage 1 leaves (0.5-1 cm sprouts); Leaf2, stage 2 leaves (2-2.5 cm leaves, not expanded); Leaf3, stage 3 leaves (expanded 7-8 cm leaves).

|  | Leaf1 | Leaf2 | Leaf3 | Petiole | Fibrous root | Callus | Germinating Seed | Flower Bud | Unopen Flower | Open Flower |
| --- | --- | --- | --- | --- | --- | --- | --- | --- | --- | --- |
| DcE2F1 | 37073,4 | 36626,4 | 42634,8 | 20569,4 | 22963,2 | 103643 | 78446,9 | 44100,1 | 41250,9 | 19539,3 |
| DcE2F2 | 76906,5 | 135295 | 269695 | 244257 | 349150 | 106600 | 97424 | 92690,4 | 277071 | 169665 |
| DcE2F3 | 132899 | 130682 | 122854 | 114693 | 59688,7 | 85931,1 | 81658,9 | 135525 | 73876,3 | 103620 |
| DcE2F4 | 38297,2 | 58295,8 | 77741,4 | 25879,9 | 76903,9 | 246725 | 101848 | 54073,2 | 36832,3 | 64138,6 |
| DcE2F5 | 258948 | 102989 | 12411 | 15331,3 | 60891,9 | 113359 | 214212 | 203065 | 19283,1 | 115664 |
| DcE2F6 | 104793 | 39259,9 | 972 | 3673,02 | 3157,02 | 8913,42 | 84481,8 | 110333 | 0 | 36492,1 |
| DcE2F7 | 319 | 0 | 1260,53 | 3803,49 | 2461,5 | 3110,08 | 1395,62 | 0 | 0 | 1734,69 |
| DcDP1 | 70347,2 | 118962 | 111696 | 142657 | 293321 | 95738,7 | 79171,2 | 67816,9 | 88343,4 | 91521,2 |
| DcDP2 | 122190 | 144221 | 90666,2 | 85677,7 | 15947,9 | 82155,7 | 121806 | 88658,4 | 218770 | 154219 |
| DcDP3 | 158226 | 233668 | 270068 | 343458 | 115515 | 153823 | 139556 | 203738 | 244574 | 243405 |
